# Identification and removal of unexpected proliferative off-target cells emerging after iPSC-derived pancreatic islet cell implantation

**DOI:** 10.1073/pnas.2320883121

**Published:** 2024-04-10

**Authors:** Hideyuki Hiyoshi, Kensuke Sakuma, Shinya Asano, Stephanie C. Napier, Shuhei Konagaya, Taisuke Mochida, Hikaru Ueno, Takeshi Watanabe, Yoshiaki Kassai, Hirokazu Matsumoto, Ryo Ito, Taro Toyoda

**Affiliations:** ^a^Takeda-CiRA Discovery and Innovation, Takeda Pharmaceutical Company Limited, Fujisawa, Kanagawa 251-8555, Japan; ^b^Takeda-CiRA Joint Program for iPS Cell Applications, Fujisawa, Kanagawa 251-8555, Japan; ^c^Axcelead Drug Discovery Partners, Inc., Fujisawa, Kanagawa 251-8555, Japan; ^d^Department of Cell Growth and Differentiation, Center for iPS Cell Research and Application, Kyoto University, Kyoto 606-8397, Japan; ^e^Drug Safety Research and Evaluation, Takeda Pharmaceutical Company Limited, Fujisawa, Kanagawa 251-8555, Japan; ^f^Department of Life Science Frontiers, Center for iPS Cell Research and Application, Kyoto University, Kyoto 606-8397, Japan

**Keywords:** induced pluripotent stem cell–derived pancreatic islet cells, cell-based therapy, type 1 diabetes mellitus, long-term safety

## Abstract

Differentiation of pancreatic endocrine cells from human pluripotent stem cells (PSCs) has been thoroughly investigated for application in cell therapy against diabetes. In the context of induced pancreatic endocrine cell implantation, previous studies have reported graft enlargement resulting from off-target pancreatic lineage cells. However, there is currently no documented evidence of proliferative off-target cells beyond the pancreatic lineage in existing studies. Here, we show that the implantation of seven-stage induced PSC-derived pancreatic islet cells (s7-iPICs) leads to the emergence of unexpected off-target cells with proliferative capacity via in vivo maturation. These cells display characteristics of both mesenchymal stem cells (MSCs) and smooth muscle cells (SMCs), termed proliferative MSC- and SMC-like cells (PMSCs). The frequency of PMSC emergence was found to be high when 10^8^ s7-iPICs were used. Given that clinical applications involve the use of a greater number of induced cells than 10^8^, it is challenging to ensure the safety of clinical applications unless PMSCs are adequately addressed. Accordingly, we developed a detection system and removal methods for PMSCs. To detect PMSCs without implantation, we implemented a 4-wk-extended culture system and demonstrated that putative PMSCs could be reduced by compound treatment, particularly with the taxane docetaxel. When docetaxel-treated s7-iPICs were implanted, the PMSCs were no longer observed. This study provides useful insights into the identification and resolution of safety issues, which are particularly important in the field of cell-based medicine using PSCs.

## Significance

The unrestricted capacity of human induced pluripotent stem cells (iPSCs) to proliferate and differentiate is a major advantage for the development of cell-based therapies. However, this advantage is accompanied by a risk of off-target cell proliferation and tumorigenesis. Therefore, minimizing the risk of off-target cell contamination is imperative for the safe use of iPSC-derived products. In this study, we identified proliferative off-target cells, termed PMSCs (proliferative mesenchymal stem cells), that emerged unexpectedly after the implantation of iPSC-derived pancreatic islet cells. Additionally, we developed an in vitro detection system and effective removal methods for PMSCs, paving the way for safer clinical applications of PSC-derived islet-like cells.

The production of tissues and organs from human pluripotent stem cells (PSCs), which have an unlimited proliferative capacity, could solve the problem of donor shortage for organ transplantation (1). Particularly, in the treatment of type 1 diabetes mellitus (T1DM), an autoimmune disorder resulting in severe loss of pancreatic β-cells, cell therapy using pancreatic endocrine cells derived from PSCs is expected to be an alternative to pancreas or islet transplantation (2, 3). Multiple studies have reported the properties and functions of induced pancreatic endocrine cells, indicating that differentiation methods are reliable and robust (4–10). In addition, these induced pancreatic endocrine cells are comparable to native human islets, showing an apparent glucose-normalizing effect with insulin secretion when implanted into T1DM model animals (4, 5, 9, 10).

Although PSC-derived differentiated cells potentially provide an unlimited supply, there is a risk of contamination with undifferentiated or off-target cells during induction (11, 12). When PSC-derived pancreatic progenitors or pancreatic endocrine cells are implanted into immunocompromised mice, graft hyperplasia caused by unintended cells in the final product has been reported (6, 10, 13, 14). These enlarged grafts primarily consist of pancreatic duct-like cystic structures that retain fluid and increase in volume more than 10-fold within 200 d of implantation (13). These cystic structures could originate from nonendocrine progenitor cells of the pancreatic lineage, which comprise 2 to 10% of the total cells as by-products (13, 15, 16). Several attempts have been made to reduce nonendocrine progenitor cells, including cell sorting and protocol optimization (6, 7, 14, 17). Although these approaches suppress cyst formation, little is known about their long-term safety.

Previously, we reported that treatment with kinase inhibitors such as PD-166866 and TR06141363 selectively and efficiently reduced nonendocrine progenitor cells (10). Using these compounds, we optimized a seven-stage stepwise differentiation protocol (4) and generated seven-stage induced PSC-derived pancreatic islet cells (s7-iPICs) (10). In our s7-iPICs, in the order of 10^4^, the proliferative nonendocrine progenitor population was reduced to undetectable levels in vitro. Additionally, when s7-iPICs were implanted into T1DM model mice in the order of 10^6^, no abnormalities were observed in the grafts, and normoglycemia was achieved. However, for clinical application in patients with T1DM, induced cells in the order of 10^8^ to 10^9^ are required (18). Therefore, we considered it necessary to investigate the long-term safety of s7-iPICs using large-scale experiments. However, implantation studies in mice generally use 10^6^ cells (4–7), and implantation at higher cell densities may result in lower cell survival and an underestimation of the risk of off-target cells. Thus, in the present study, we performed multiple experiments with s7-iPICs in the order of 10^6^ to evaluate cumulative cells in the order of 10^8^ or higher. Consequently, we observed the appearance of unexpected proliferative off-target cells, which were different from cells constituting the pancreatic duct-like cystic structures. As the unexpected cells could pose a major risk for clinical applications, the purpose of this study was to identify the characteristic profiles of this off-target cell population to propose an effective detection system and reduction strategy.

## Results

### Unexpected Abnormal Outgrowth in s7-iPIC Grafts Is Outside the Pancreatic Lineage and Continues to Proliferate after Implantation.

**Fig. 1.**
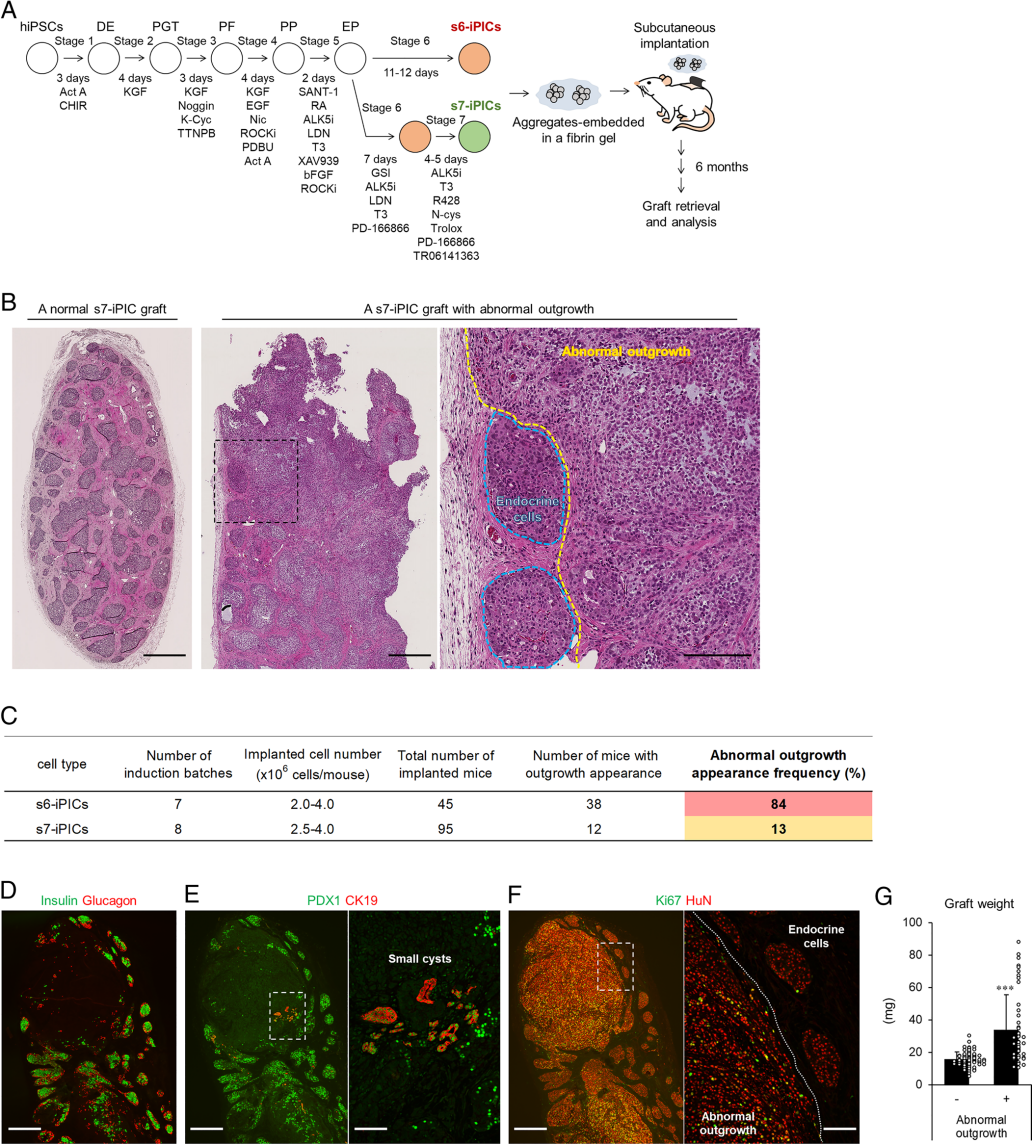
Unexpected abnormal outgrowth in s7-iPIC grafts is outside the pancreatic lineage and continues to proliferate after implantation. (*A*) Schematic representation of s6-iPIC and s7-iPIC differentiation and subcutaneous implantation using fibrin gel. DE, definitive endoderm; PGT, primitive gut tube; PF, posterior foregut; PP, pancreatic progenitors; EP, endocrine progenitors; Act A, activin A; CHIR, CHIR99021; K-Cyc, KAAD-cyclopamine; Nic, nicotinamide; ROCKi, ROCK inhibitor; RA, retinoic acid; ALK5i, ALK5 inhibitor II; LDN, LDN-193189; GSI, γ-secretase inhibitor; N-cys, *N*-acetyl cysteine. (*B*–*G*) Cell implantation experiments in streptozotocin-injected and normal NOD-scid mice. Mice were implanted with s6-iPICs or s7-iPICs (2.0 to 4.0 × 10^6^ cells/mouse) embedded in a fibrin gel into the subcutaneous space. (*B*) Hematoxylin and eosin (HE)-stained sections 24 wk after implantation. The *Left* image shows a normal s7-iPIC graft, and the two *Right* images show a s7-iPIC graft with abnormal outgrowth. The high-magnification image is an enlarged image of the area enclosed by the dotted line in the low-magnification image. Black scale bars indicate 500 μm at low magnification and 200 μm at high magnification. The images are representative of dozens of samples showing similar results. (*C*) Details of abnormal outgrowth appearance frequency after s6-iPIC and s7-iPIC implantation. (*D*–*F*) Immunohistochemical images of an s7-iPIC graft with abnormal outgrowth at 24 wk postimplantation. White scale bars indicate 500 μm at low magnification and 100 μm at high magnification. HuN; human nucleus. Images were taken from serial sections of the same sample and are representative of dozens of samples showing similar results. (*G*) Graft weight of samples without abnormal outgrowth (n = 82, combined total number of s6-iPIC and s7-iPIC grafts); and samples with abnormal outgrowth (n = 47, combined total number of s6-iPIC and s7-iPIC grafts). See *SI Appendix*, Fig. S1*A* for graft weight distribution according to the type and number of implanted cells. Data are shown as the mean ± SD. ***P < 0.001, Aspin–Welch test.

To evaluate the long-term safety profile of s7-iPICs on a large scale, we repeated experiments in which fibrin gel–embedded s7-iPICs in the order of 10^6^ were subcutaneously implanted into immunodeficient mice (Fig. 1*A*). Similarly, we also evaluated s6-iPICs, which were cultured for the same period under stage 6 conditions instead of receiving stage 7 treatment. At 23 to 26 wk after implantation, most s7-iPIC grafts were composed of endocrine cell clusters, host-derived blood vessels, and host-derived fibrous tissue, and did not show abnormal cell proliferation (Fig. 1*B*). However, s7-iPIC grafts with unexpected abnormal outgrowth were infrequently observed (13%, 12/96 mice) (Fig. 1 *B* and *C*). In addition, s6-iPIC grafts showed abnormal outgrowth more frequently than s7-iPIC grafts (84%, 38/45 mice) (Fig. 1*C*). Although the composition of the cell population was the same in s6-iPIC and s7-iPIC at single-cell resolution, the content ratio of each population was different (10). In s6-iPICs, the content ratio of the nonendocrine progenitor cell population was reduced by PD-166866 treatment but was slightly higher than that in s7-iPICs. Therefore, the frequency of abnormal outgrowth appears to be associated with the degree of residual nonendocrine progenitor cells.

To characterize the abnormal outgrowth, immunostaining was performed; however, neither pancreatic endocrine cell markers (PDX1, insulin, and glucagon) nor markers of pancreatic duct-like cystic structures (PDX1 and CK19) were detected (Fig. 1 *D* and *E*). In contrast, the abnormal outgrowth was stained with a human nuclear antibody (HuN), indicating that the structure was derived from the implanted s7-iPICs, and expressed the proliferation marker Ki67 at a high frequency (Fig. 1*F*). Furthermore, several grafts with abnormal outgrowth weighed more than threefold (>60 mg) the average weight (15.8 ± 4.4 mg) of the grafts without abnormal outgrowth (Fig. 1*G* and *SI Appendix*, Fig. S1*A*). These results suggest that unexpected cells constituting the outgrowth are outside the pancreatic lineage and that they continue to proliferate and gradually become apparent after implantation. Although the frequency of abnormal outgrowth in s7-iPIC grafts appeared to be relatively low (13%, 12/96 mice) (Fig. 1*C*), this was the frequency when implanted in the order of 10^6^; and as cells in the order of 10^8^ to 10^9^ are required for patients with T1DM (18), this abnormal proliferation is assumed to be a major risk for clinical application.

### The Unknown Cell Population that Cannot Be Classified as Known Cell Types in Single-Cell Analysis Could Be Responsible for Abnormal Outgrowth.

**Fig. 2.**
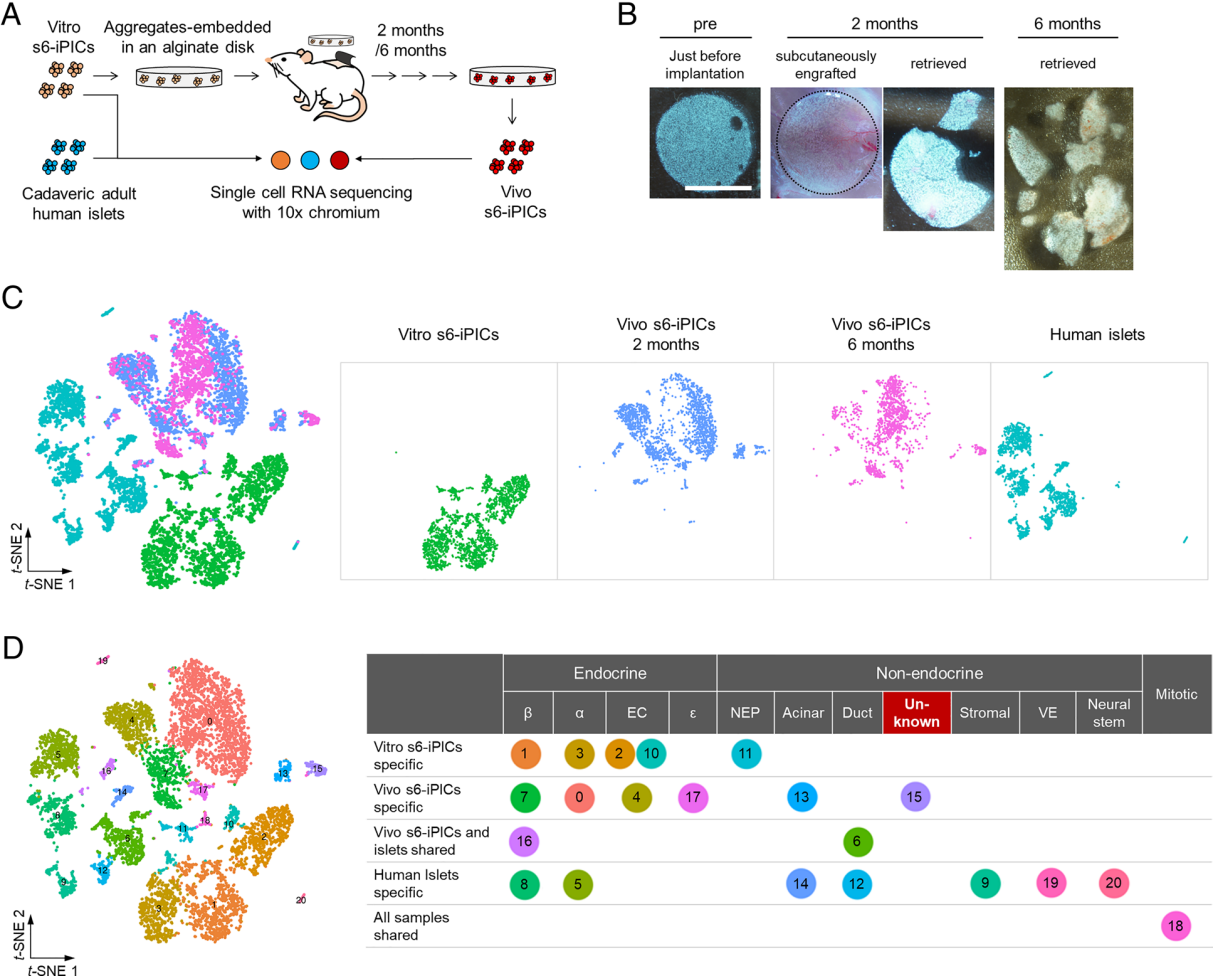
The unknown cell population that cannot be classified as known cell types in single-cell analysis could be responsible for abnormal outgrowth. (*A*) Schematic representation of vitro and vivo s6-iPIC samples subjected to scRNA-seq. (*B*) Macroscopic photographs of vivo s6-iPIC samples. From *Left* to *Right*, alginate gel–embedded s6-iPICs before implantation, the graft was subcutaneously engrafted or retrieved 2 mo after implantation, and the graft was retrieved 6 mo after implantation. The area surrounded by a dotted line indicates the area where implanted cells have engrafted subcutaneously. The white bar indicates 5 mm. (*C*) Cell distribution on *t*-SNE projections for four combined samples and each sample. The four samples were broken down as follows: one sample of vitro s6-iPICs, two samples of vivo s6-iPICs (2 and 6 mo after implantation), and one sample of reference human islets. (*D*) Shared nearest neighbor clustering identified five clusters specific to Vitro s6-iPICs (1, 2, 3, 10, and 11), six clusters specific to vivo s6-iPICs (0, 4, 7, 13, 15, and 17), and seven clusters specific to human islets (5, 8, 9, 12, 14, 19, and 20). Clusters 6 and 16 were shared by vivo s6-iPICs and human islets. Cluster 18, a population of proliferative cells, was shared by all the samples. See *SI Appendix*, Fig. S2*C* for further details. Each cluster was assigned to a known cell type using the characteristic gene expression shown in *SI Appendix*, Fig. S2*D*. β, β-fate cells or actual β-cells; α, α-fate cells or actual α-cells; EC, enterochromaffin-fate cells; ε, ε-fate cells; NEP, nonendocrine progenitor cells; Acinar, acinar-like cells or actual acinar cells; Duct, duct-like cells or actual duct cells; Unknown, unknown cells that could not be assigned to known cells; Stromal, stromal cells; VE, vascular endothelial cells; Neural stem, neural stem cells; Mitotic, Mitotic cells.

To understand and eliminate abnormal outgrowth, we attempted to extract and profile the cells constituting abnormal outgrowth using single-cell RNA sequencing (scRNA-seq). Considering the infrequency of abnormal outgrowth in s7-iPIC grafts, we used s6-iPICs to increase the possibility of obtaining the cells responsible for abnormal outgrowth (Fig. 2*A*). In addition, to prevent reduction in sequencing depth due to contamination by host-derived cells, we embedded s6-iPICs in alginate gel, which is a nonbiodegradable material (19, 20). Two and six months after subcutaneous implantation, the grafts could be separated from the host mouse while maintaining the shape of the alginate gel (Fig. 2*B*). In these in vivo samples, the number of cells excluded during quality control by the mitochondrial gene ratio was limited, and host cell contamination was minimal (*SI Appendix*, Fig. S2 *A* and *B*). To extract the characteristics of the in vivo samples, we combined their data with sequence data from the cells immediately before implantation (Vitro s6-iPICs) and from adult human islets as references, and performed nonbiased cluster classification (Fig. 2 *A*, *C*, and *D*). As there was little difference in the cluster classifications of the in vivo samples at 2 and 6 mo postimplantation (*SI Appendix*, Fig. S2*C*), the two in vivo samples were collectively treated as Vivo s6-iPICs.

Based on previous reports (7, 8, 15), we assigned each cluster to a known cell type using characteristic gene expression as an indicator (Fig. 2*D* and *SI Appendix*, Fig. S2*D*). As a candidate cell population responsible for abnormal outgrowth, cluster 18 initially caught our attention because it had the highest expression of the proliferation marker *MKI67* (*SI Appendix*, Fig. S2*D*). However, cluster 18 included cells from all samples, including human islets, and the majority of cells in cluster 18 had high S.Score and G2M.Score in cell cycle phase assignment analysis (*SI Appendix*, Fig. S2 *C* and *E*). Therefore, we concluded that cluster 18 was a heterogeneous population of mitotic cells (Fig. 2*D*) and assumed that it would be difficult to extract the cells responsible for abnormal outgrowth from an in-depth analysis of cluster 18. Hence, we focused on clusters specific to Vivo s6-iPICs. Among the clusters derived from Vivo s6-iPICs, five (clusters 0, 4, 7, 16, and 17) were classified as endocrine cells, and three (clusters 6, 13, and 15) were classified as nonendocrine cells. In the three nonendocrine cell clusters, clusters 6 and 13 were pancreatic duct-like cells expressing *KRT19*, *HIF1B*, and *SOX9*, and pancreatic acinar-like cells expressing *CTRB1* and *CTRB2*, respectively (*SI Appendix*, Fig. S2*D*). Such pancreatic exocrine cell clusters were also naturally present in adult human islets (clusters 6, 12, and 14) (Fig. 2*D* and *SI Appendix*, Fig. S2*D*). In contrast, cluster 15 showed no expression of pancreatic lineage markers and expressed MSC-related genes (7), such as *VIM*, *COL3A1*, and *COL1A1* (*SI Appendix*, Fig. S2*D*). In addition, cluster 15 contained several cells with high S.Score and G2M.Score (*SI Appendix*, Fig. S2*E*), leading us to hypothesize that cluster 15 is responsible for abnormal outgrowth after implantation.

### Abnormal Outgrowth Consists of Proliferative MSC- and SMC (Smooth Muscle Cells)-Like Cells (PMSCs).

**Fig. 3.**
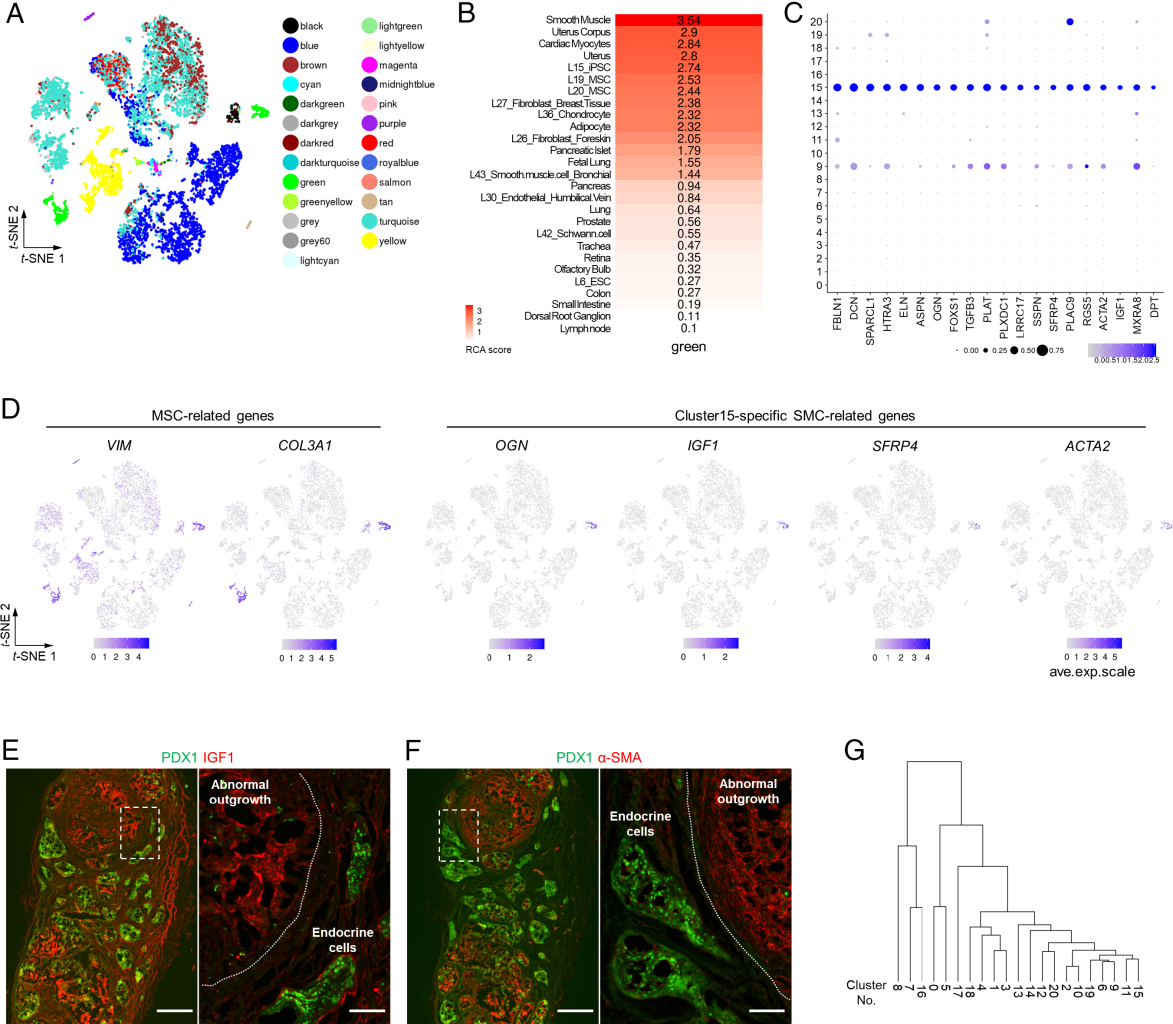
Abnormal outgrowth consists of proliferative MSC- and SMC-like cells (PMSCs). (*A*) Reclassified cell populations identified by RCA on the t-SNE projection. (*B*) Heatmap of tissues and cell lines with similarity to cells annotated as green (clusters 15 and 9 in Fig. 2*D*) in the RCA. See *SI Appendix*, Fig. S3*A* for a full RCA heatmap. (*C*) Bubble plot of the top 20 differentially expressed genes in cluster 15 (*P* < 0.05, fold-change > 1.2, and pct.2 < 0.05). Color intensity indicates average relative expression levels. The bubble size indicates the percentage of expressing cells. (*D*) Single-cell gene expression of MSC- and cluster 15–specific SMC-related markers on the t-SNE projection. (*E* and *F*) Immunohistochemical images of an s7-iPIC graft with abnormal outgrowth at 24 wk postimplantation. White scale bars indicate 500 μm at low magnification and 100 μm at high magnification. Images were taken from serial sections of the same sample as in Fig. 1 *D*–*F* and are representative of dozens of samples showing similar results. See *SI Appendix*, Fig. S6*A* for immunohistochemical data on other PMSC-related markers. (*G*) Hierarchical clustering analysis. Cluster 15 was most closely related to the nonendocrine progenitor cells in vitro s6-iPICs (cluster 11) and most distantly related to the β-cell population (clusters 8, 7, and 16).

To scrutinize the characteristics of cluster 15, we performed reference component analysis (RCA), which indicates transcriptome similarities with known tissues or cell lines (21) (Fig. 3*A*). Cluster 15 was classified as “green” and had high scores for smooth muscle, uterus, MSCs, and iPSCs (induced PSCs) (Fig. 3 *A* and *B* and *SI Appendix*, Fig. S3*A*). As stromal cells in adult human islets (cluster 9) also belonged to the green group and showed MSC-related marker expression (*SI Appendix*, Fig. S2*D*), we proceeded with our analysis by comparing these two clusters. To extract the differences between clusters 15 and 9, we estimated the pseudotime from cluster 15 to cluster 9 (*SI Appendix*, Fig. S4*A*). In addition, we calculated gene clusters composed of genes with common pseudotime expression kinetics and performed gene ontology term analysis (*SI Appendix*, Fig. S4 *B* and *C*). Compared to cluster 9, cluster 15 had relatively active pathways related to developmental processes (gene cluster 1), gene transcription processes (gene cluster 2), and mitotic cell cycle transition/catabolic processes (gene cluster 3). These results suggest that cluster 15 is an immature and proliferative population, unlike true stromal cells (cluster 9).

We extracted the top 20 differentially expressed genes that were highly expressed in cluster 15 but minimally expressed in the other clusters (Fig. 3*C*). These 20 genes were then inputted into an external database, TissueEnrich (https://tissueenrich.gdcb.iastate.edu/) (22), and the uterus-related genes *OGN*, *IGF1*, and *SFRP4* were observed to be included (*SI Appendix*, Fig. S5 *A* and *B*). We validated the expression of *OGN*, *IGF1*, and *SFRP4* on the *t*-SNE projections and observed that these uterus-related genes were specifically expressed in cluster 15, whereas MSC-related genes, such as *VIM* and *COL3A1*, were not only expressed in cluster 15 but also in cluster 9 (Fig. 3*D*). In addition, cluster 15 specifically expressed the gene for α-smooth muscle actin (α-SMA), *ACTA2*, possibly reflecting smooth muscle traits (Fig. 3 *C* and *D*). Tissue immunostaining was performed to evaluate whether the cluster 15–specific markers were expressed at the protein level. IGF1, α-SMA, and SFRP4 proteins were confirmed to be expressed in the abnormal outgrowth, together with MSC-related proteins Vimentin and Collagen III (Fig. 3 *E* and *F* and *SI Appendix*, Fig. S6*A*). Based on the results thus far, we concluded that the cell population in cluster 15 constitutes the abnormal outgrowth emerging after implantation. This population exhibited traits associated with MSCs and SMCs, including uterine characteristics. Accordingly, this in vivo population is hereafter referred to as proliferative MSC- and SMC-like cells (PMSCs).

We attempted to demonstrate that PMSCs are not a cell type specific to our iPICs by analyzing previously reported data. We highlighted a previous report that provided scRNA-seq data using in vivo grafts of islet-like cells derived from ES and iPS cell lines, which are different from the cell lines of our iPICs (8). Reanalysis of the scRNA-seq data in this report revealed the presence of a cell population expressing PMSC-specific markers such as *VIM*, *COL3A1*, *OGN*, *IGF1*, *SFRP4*, and *ACTA2* in the grafts (*SI Appendix*, Fig. S7 *A* and *B*). Remarkably, this in vivo graft, including a PMSC-equivalent cell population, was not only derived from cell lines different from our iPICs but also differed in the detailed induction and implantation methods (8). Hence, PMSCs are not a phenomenon specific to our cell line, induction protocol, or implantation method but are commonly present in the grafts of PSC-derived islet-like cells.

Notably, the PMSC-specific markers were not expressed before implantation (vitro s6-iPICs) (Fig. 3*D*). To evaluate the origin of PMSCs, we conducted a hierarchical clustering analysis. Cluster 11 was identified as the nearest neighbor of PMSCs (cluster 15) (Fig. 3*G*). Cluster 11 is the nonendocrine progenitor population, which is included in vitro s6-iPICs as a by-product (Fig. 2*D*), most of which are expected to mature into pancreatic duct-like cells (cluster 6) that form cysts after implantation (7, 8, 10, 13, 23). Hierarchical clustering analysis suggested that cells in the nonendocrine progenitor population could not only mature into cysts but also into PMSCs. This hypothesis is consistent with the higher frequency of PMSCs in s6-iPICs (Fig. 1*C*), which contain more nonendocrine progenitor cells than s7-iPICs (10).

### An EGF (Epidermal Growth Factor)-Supplemented Extended Culture System Exposes a Cell Population (Putative PMSCs) that Closely Resembles In Vivo PMSCs.

**Fig. 4.**
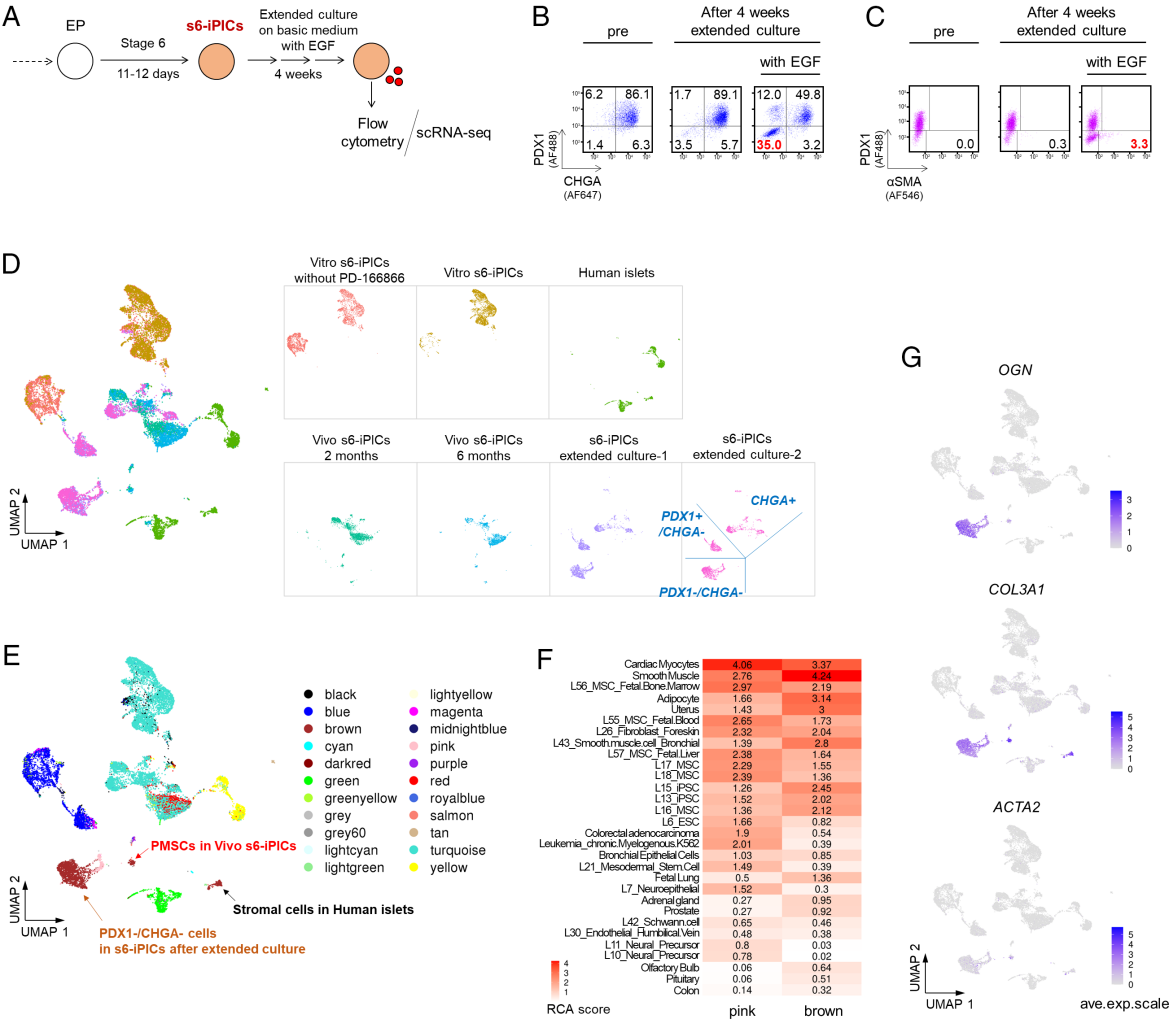
An EGF-supplemented extended culture system exposes a cell population (putative PMSCs) that closely resembles in vivo PMSCs. (*A*) Schematic representation of extended culture, subsequent flow cytometry analysis, and scRNA-seq using s6-iPICs. (*B* and *C*) Representative flow cytometry plots illustrating the protein expression of s6-iPICs before and after extended culture. The numbers in each plot diagram show the percentage of each population. The reproducibility was confirmed in three independent experiments. (*D*) Cell distribution on uniform manifold approximation and projections (UMAPs) for seven combined samples and for each sample. We reanalyzed the four samples shown in Fig. 2*C* with the addition of the following three samples: one sample of vitro s6-iPICs cultured without PD-166866 and two samples (technical duplicates) of s6-iPICs after 4 wk of extended culture including EGF treatment. Cells in the samples after extended culture were classified into three types based on gene expression intensity: *PDX1*^−^/*CHGA*^−^, *PDX1*^+^/*CHGA*^−^, and *CHGA*^+^ populations. See *SI Appendix*, Fig. S9 *A*–*C* for further details. (*E*) Newly classified cell populations identified by RCA on the UMAP. The *PDX1*^−^/*CHGA*^−^ population in the extended culture samples, PMSCs in Vivo s6-iPICs, and stromal cells in human islets were classified as “pink” and “brown”. *SI Appendix*, Fig. S9 *A* and *B* shows the position of PMSCs in vivo s6-iPICs and stromal cells in human islets on UMAPs. (*F*) Heatmap of tissues and cell lines similar to cells annotated as pink and brown in the RCA. See *SI Appendix*, Fig. S9*D* for a full RCA heatmap. (*G*) Single-cell gene expression of PMSC markers on the UMAP.

The analyses thus far suggest that PMSCs likely originate from nonendocrine progenitor cells; however, their fate is not determined prior to implantation, and PMSCs only become apparent after several months of in vivo maturation. Thus, the exploration of PMSC removal methods requires implantation trials and months of growth and maturation, which results in an extremely low throughput. Therefore, we were challenged to develop a detection system that allows the prediction of the appearance of PMSCs without relying on implantation. Because an extended culture of preimplanted cells in vitro can mimic the in vivo maturation of pancreatic endocrine cells to some extent (7, 24), we considered that extended culture might induce cells with a profile similar to that of PMSCs (Fig. 4*A*). However, extended culture of s6-iPICs for 4 wk in a simple basal medium resulted in dominant chromogranin A (CHGA)-positive endocrine cells and no obvious increase in off-target cells (Fig. 4*B*). We noted that the addition of EGF receptor ligands, such as betacellulin and EGF, to the s6-iPIC-induction process, resulted in a dose-dependent increase in the CHGA-negative nonendocrine population, which is likely the origin of PMSCs (*SI Appendix*, Fig. S8 *A* and *B*). Accordingly, we attempted an extended culture with EGF supplementation and observed a marked increase in the PDX1^−^/CHGA^−^ population (Fig. 4*B*). Furthermore, the PDX1^−^/CHGA^−^ population contained cells expressing α-SMA, a PMSC-specific marker (Fig. 4*C*).

To examine the relationship between the PDX1^−^/CHGA^−^ population and in vivo PMSCs, we reanalyzed the scRNA-seq data by adding sequence data from EGF-supplemented extended culture samples. Similar to the flow cytometry results, the cells after extended culture could be classified into three types: *PDX1*^−^/*CHGA*^−^, *PDX1*^+^/*CHGA*^−^, and *CHGA*^+^ (Fig. 4*D* and *SI Appendix*, Fig. S9 *A*–*C*). By performing RCA, we observed that the *PDX1*^−^/*CHGA*^−^ population after extended culture scored highly for smooth muscle, uterus, and MSCs, as well as in vivo PMSCs (Fig. 4 *E* and *F* and *SI Appendix*, Fig. S9*D*). In addition, the *PDX1*^−^/*CHGA*^−^ population characteristically expressed in vivo PMSC-related genes such as *OGN* and *COL3A1*, and a portion of the population expressed *ACTA2* (Fig. 4*G*). These results suggest that the PDX1^−^/CHGA^−^ population that emerges after EGF-supplemented extended culture is almost equivalent to the PMSCs that appear after implantation.

### Cisplatin and Docetaxel Effectively Remove Putative PMSCs through Mechanisms Other than Kinase Inhibition.

**Fig. 5.**
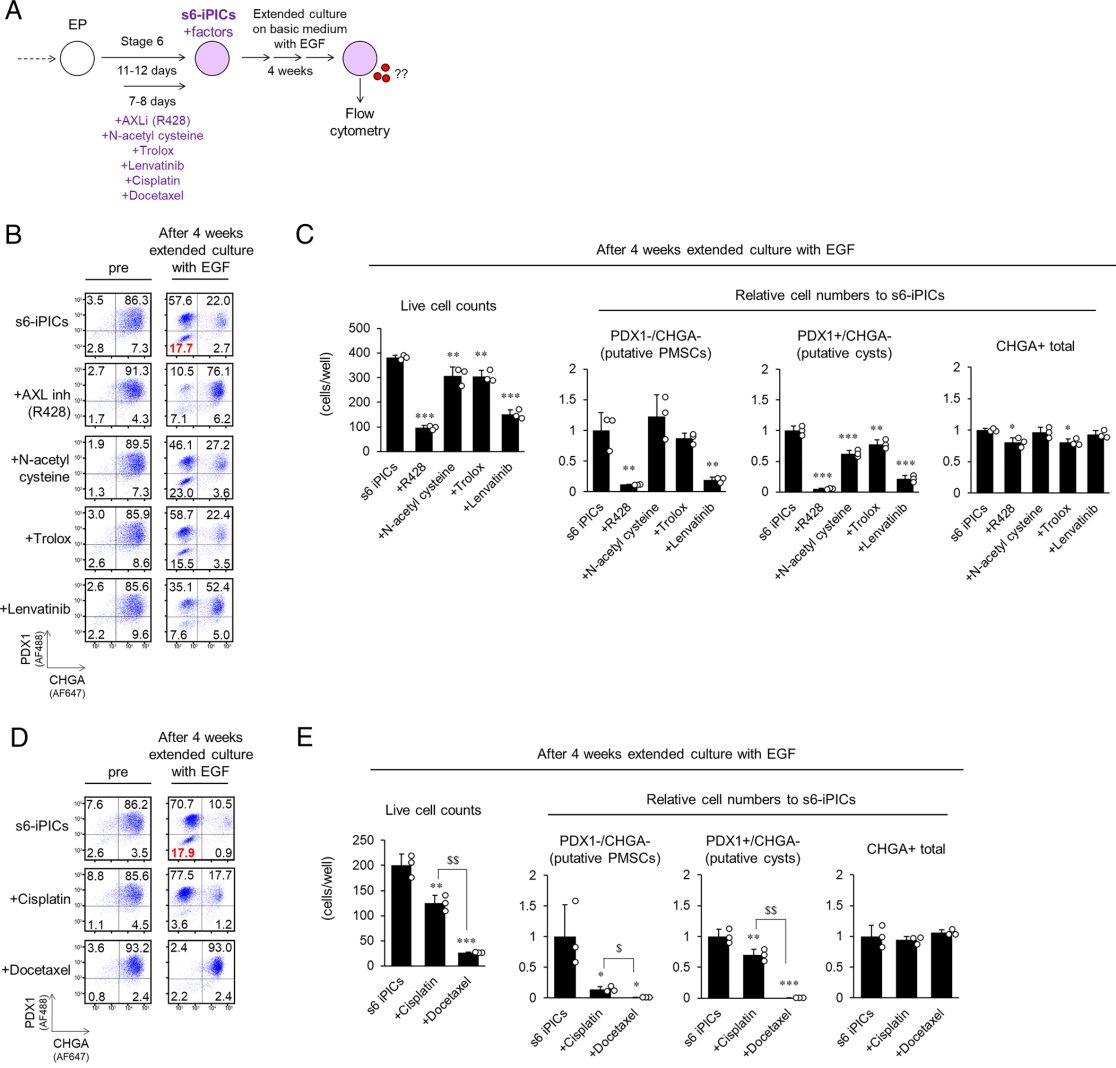
Cisplatin and docetaxel effectively remove putative PMSCs through mechanisms other than kinase inhibition. (*A*) Schematic representation of s6-iPIC derivatives induced with or without additional compound treatment and subsequent extended culture and flow cytometry analysis. (*B*–*E*) Representative flow cytometry plots illustrating protein expression before and after extended culture of s6-iPIC derivatives (*B* and *D*). Live cell counts and relative cell numbers of PDX1^−^/CHGA^−^, PDX1^+^/CHGA^−^, and CHGA^+^ populations in each s6-iPIC derivative postextended culture. The number of cells in control s6-iPICs was set to “1” (*C* and *E*). The number of cells in each population was calculated from flow cytometry results (percentage of each population) and live cell counts. Data are shown as the mean ± SD (n = 3, technical replicates). The reproducibility of the effect of docetaxel was confirmed in three independent experiments. *P < 0.05, **P < 0.01, and ***P < 0.001 vs. s6-iPICs, Dunnett’s test. ^$^*P* < 0.05, ^$$^*P* < 0.01, Aspin–Welch test.

As EGF-supplemented extended culture enabled us to detect putative PMSCs (PDX1^−^/CHGA^−^ population) without implantation, we sought ways to remove putative and in vivo PMSCs. We noted that s7-iPICs had a lower frequency of in vivo PMSCs than s6-iPICs (Fig. 1*C*). Therefore, we evaluated the cells generated by adding the s7-iPIC-specific inducers R428, N-acetylcysteine, and Trolox to s6-iPICs (Fig. 5*A*). Although the addition of N-acetylcysteine and Trolox did not change the population after extended culture, the addition of R428, an AXL inhibitor, significantly reduced the putative PMSCs (Fig. 5 *B* and *C*). Since AXL inhibitors are kinase inhibitors and PMSCs are a proliferative cell population with uterine characteristics, we examined a multikinase inhibitor, lenvatinib, which is effective against endometrial carcinoma (25). Lenvatinib induced a similar reduction in putative PMSCs to R428 (Fig. 5 *B* and *C*). These results suggest that kinase inhibitors can reduce in vivo PMSCs and that factors that reduce in vivo PMSCs can be detected using extended culture. However, in vivo PMSCs appeared even in s7-iPICs that were treated with the three kinase inhibitors R428, PD-166866, and TR06141363. Thus, a mechanism of action other than kinase inhibition is necessary to ensure the removal of residual in vivo PMSCs from s7-iPIC grafts.

Considering that PMSCs have proliferative abilities and uterine characteristics, we focused on the platinum complex, cisplatin, and taxanes, docetaxel and paclitaxel, which are used as first-line chemotherapies for endometrial carcinoma (26, 27). In cells induced with cisplatin or docetaxel treatment, we observed a significant reduction in the number of putative PMSCs (Fig. 5 *D* and *E*). Docetaxel exhibited a more pronounced putative PMSC removal than cisplatin (Fig. 5*E*). In addition, docetaxel clearly reduced the PDX1^+^/CHGA^−^ population (putative cysts), which is the other population that increases after extended culture and the pancreatic lineage nonendocrine population that can form cysts after implantation. Conversely, cisplatin and docetaxel treatments did not affect the number of CHGA^+^ endocrine cells (Fig. 5*E*), suggesting that the percentage increase in the PDX1^−^/CHGA^−^ and PDX1^+^/CHGA^−^ populations after extended culture (Fig. 5*D*) is due to proliferation of nonendocrine cell population rather than a shift from the CHGA^+^ endocrine cell population. We also evaluated other chemotherapeutic agents, such as the alkylating agent, cyclophosphamide; the antiestrogenic agents, tamoxifen and anastrozole; and the HER1/HER2 inhibitor, lapatinib. However, they had little effect on putative PMSC and cyst populations (*SI Appendix*, Fig. S10 *A* and *B*). These results indicate that platinum complexes and taxanes are effective compounds for removing in vivo PMSCs via a mechanism other than kinase inhibition. In addition, the compounds that appeared to be effective in removing PMSCs had little effect or tended to increase the insulin^+^/NKX6.1^+^ and insulin^+^/NKX6.1^−^ populations, which were likely to mature into β- and α-cells after implantation (*SI Appendix*, Figs. S11 *A*–*C* and S12 *A* and *B*).

### Docetaxel Treatment of s7-iPICs Abrogates the Appearance of Off-Target Cells while Showing Therapeutic Efficacy.

**Fig. 6.**
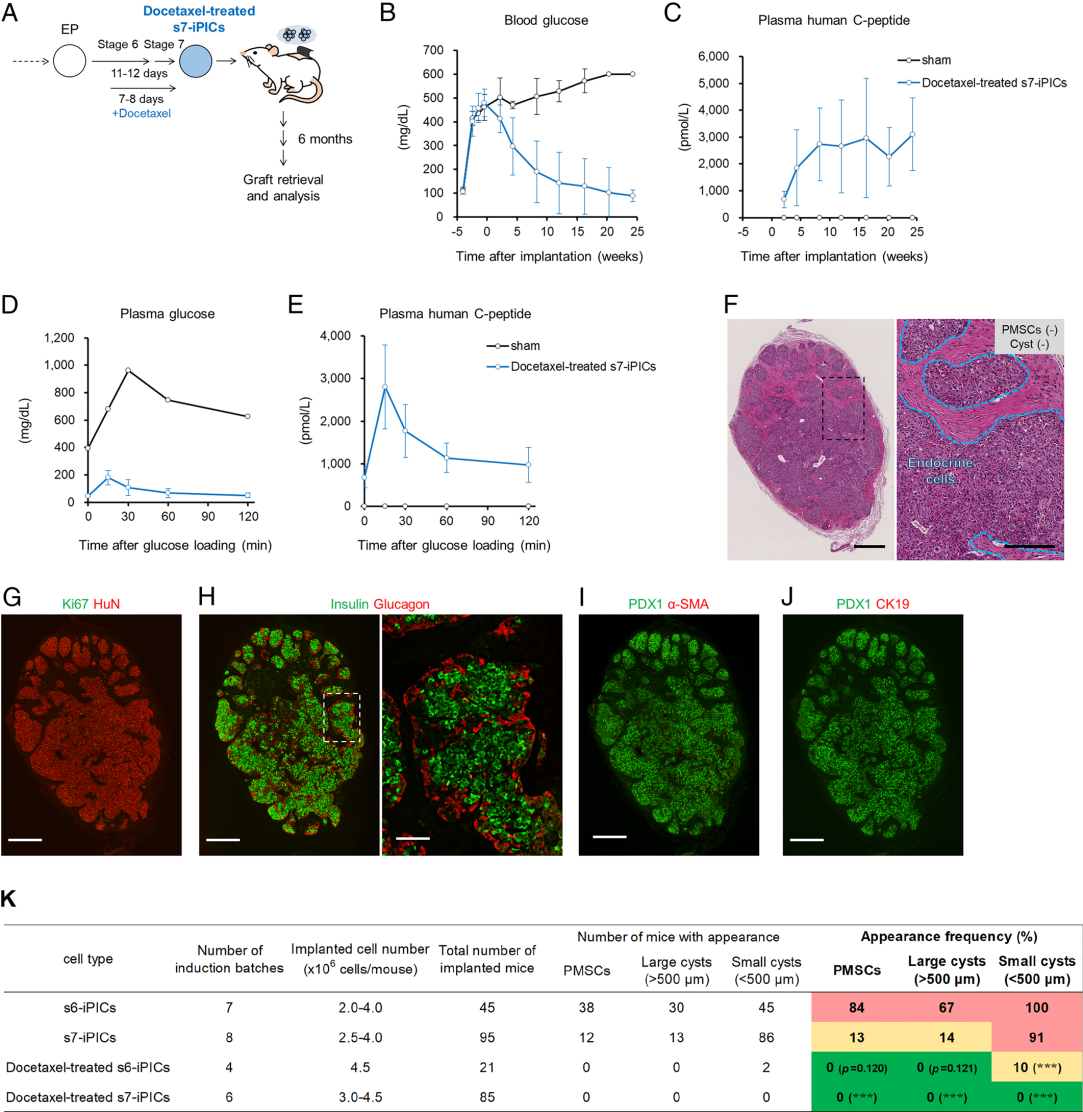
Docetaxel treatment of s7-iPICs abrogates the appearance of off-target cells while showing therapeutic efficacy. (*A*) Schematic representation of docetaxel-treated s7-iPIC differentiation and subcutaneous implantation using fibrin gel. (*B*–*J*) Cell implantation experiments in streptozotocin-injected NOD-scid mice. Mice were implanted with docetaxel-treated s7-iPICs (3.0 to 4.5 × 10^6^ cells/mouse) embedded in a fibrin gel into the subcutaneous space. (*B* and *C*) Blood glucose and plasma human C-peptide levels after docetaxel-treated s7-iPIC (4.0 × 10^6^ cells/mouse) implantation. Data are shown as mean ± SD (sham: n = 5→1, docetaxel-treated s7-iPICs: n = 18→13). The decrease in the n number was caused by unexpected death. See *SI Appendix*, Fig. S12 *A*–*D* for efficacy data of the other iPICs. (*D* and *E*) Plasma glucose, and human C-peptide levels during the oral glucose tolerance test 23 wk after docetaxel-treated s7-iPIC (4.0 × 10^6^ cells/mouse) implantation. Data are shown as mean ± SD (sham: n = 1, docetaxel-treated s7-iPICs: n = 13). (*F*) HE-stained sections 24 wk after implantation. Black scale bars indicate 500 μm at low magnification and 200 μm at high magnification. The images are representative of dozens of samples showing similar results. (*G*–*J*) Immunohistochemical images of a docetaxel-treated s7-iPIC graft at 24 wk postimplantation. White scale bars indicate 500 μm at low magnification and 100 μm at high magnification. HuN; human nucleus. Images were taken from serial sections of the same sample and are representative of dozens of samples showing similar results. See *SI Appendix*, Fig. S12 *E*–*H* for HE and immunohistochemical images on other iPICs. (*K*) Details of PMSC, large cyst (>500 μm in diameter), and small cyst (<500 μm in diameter) appearance frequency after fibrin gel–embedded subcutaneous implantation of s6-iPIC, s7-iPIC, docetaxel-treated s6-iPIC, and docetaxel-treated s7-iPIC. The parts of s6-iPICs and s7-iPIC data are previously shown in Fig. 1*C* and are repeated here for easy comparison between all groups. *P < 0.05, **P < 0.01, and ***P < 0.001 vs. s7-iPICs, Fisher’s exact test.

Finally, we determined whether the putative PMSC and cyst removal effects of docetaxel were effective in vivo in combination with s7-iPICs (Fig. 6*A*). Similar to the results obtained after implanting s7-iPICs without docetaxel treatment (10) (*SI Appendix*, Fig. S12 *C* and *D*), streptozotocin-induced diabetic mice implanted with docetaxel-treated s7-iPICs showed human C-peptide levels of >2,000 pmol/L (>6 ng/mL) in the plasma within 8 wk of implantation. Accordingly, the blood glucose levels in these mice were normalized (Fig. 6 *B* and *C*). To evaluate insulin secretion in response to glucose levels, an oral glucose tolerance test was performed 23 wk postimplantation. Plasma human C-peptide levels increased within 15 min of glucose loading (Fig. 6 *D* and *E*). Immunohistochemical evaluation of grafts revealed that most HuN-positive implanted cells were insulin- and glucagon-positive pancreatic endocrine cells, with neither α-SMA-positive PMSCs nor CK19-positive cysts observed 24 to 30 wk after implantation (Fig. 6 *F*–*J*). We repeated the same experiment, finally implanting docetaxel-treated s7-iPICs in 85 mice (cumulative implanted cell number > 3.0 × 10^8^ cells) and observed neither PMSCs nor cysts (Fig. 6*K* and *SI Appendix*, Fig. S12 *E*–*H*). To clarify the effect of docetaxel alone in vivo, we evaluated docetaxel-treated s6-iPICs. Docetaxel treatment resulted in a reduction in PMSC and cyst frequency (s6-iPICs vs. docetaxel-treated s6-iPICs), which was more evident than the effect of s7 factors (s6-iPICs vs. s7-iPICs) (Fig. 6*K*). In summary, docetaxel treatment is not only effective in in vitro extended culture but also in in vivo implantation. Furthermore, when combined with s7-iPICs, docetaxel can significantly reduce the risk of off-target cell appearance after implantation while maintaining therapeutic efficacy.

## Discussion

In the present study, we identified PMSCs, characterized by MSC and SMC traits, as unexpected proliferative off-target cells that emerged after the implantation of PSC-derived islet-like cells (Figs. 1–3). As PMSCs are expected to appear at a high frequency when s7-iPICs are implanted in the order of 10^8^, we assumed that their clinical application would be challenging without addressing these PMSCs. However, the detection and removal of in vivo PMSCs was difficult because of the following reasons: i) PMSC appearance is infrequent in implantation studies using cells on the order of 10^6^, especially with s7-iPICs (Fig. 1*C*); and ii) Detection of PMSCs takes several months because PMSCs do not express their specific markers before implantation and only become apparent after several months of in vivo maturation (Fig. 3*D*). Therefore, we developed a 4-wk-extended culture system and exposed putative PMSCs (PDX1^−^/CHGA^−^ population) without implantation (Fig. 4). Moreover, we demonstrated that putative and in vivo PMSCs could be reduced by compound treatment, particularly with the taxane docetaxel (Figs. 4 and 5). In fact, in mice implanted with docetaxel-treated s7-iPICs, the frequency of in vivo PMSC appearance was suppressed, whereas the antidiabetic activity remained clear (Fig. 6).

Cells equivalent to in vivo PMSCs were present in grafts of ESC/iPSC-derived islet-like cells induced in a previous study (8) (*SI Appendix*, Fig. S7). Another study showed proliferative cells with MSC-related gene expression observed during scRNA-seq using samples obtained from extended cultures of ESC-derived islet-like cells (7). Therefore, we do not regard PMSCs as a cell type specific to our iPICs. The lack of attention paid to PMSCs could be due to the dominance of cyst formation in situations involving abundant residual nonendocrine progenitor cells (6, 13, 14); thus, PMSCs in the grafts could have been overlooked. In fact, the presence of PMSCs was not detected in our early implantation experiments using cells with a large number of nonendocrine progenitors, as rapid graft enlargement caused by pancreatic duct-like cysts was observed (10). In this study, in vivo PMSCs were detected because of the following reasons: i) the use of s6-iPICs and s7-iPICs, in which nonendocrine progenitors were markedly reduced by our original modification, suppressed cyst formation; and ii) repeated implantation experiments allowed us to evaluate a large number of cells. As several clinical trials have been initiated using cells containing off-target cells that lead to cyst formation (13, 28), clinical application would be possible without the complete removal of off-target cells. For example, the risk to off-target cells can be significantly reduced by limiting their distribution using robust devices (28). However, because the risk of device breakage due to physical impact or other factors cannot be completely eliminated, the number of off-target cells should be reduced as much as possible. In this study, we successfully removed off-target cells, which paved the way for the safe and long-term implantation of PSC-derived islet-like cells.

Possible approaches for removing off-target cells contaminating PSC-derived cells include cell sorting (6, 7), metabolic selection (29–31), and compound treatment (10, 14, 17). Among these approaches, we selected compound treatment, which can easily process more than 10^8^ in vitro-generated cells. The kinase inhibitors, cisplatin, and docetaxel were identified as compounds that are effective at reducing putative PMSCs through different mechanisms of action (Fig. 5). However, these compounds are not novel from the perspective of elimination approaches for proliferating cells. In contrast, in the process of identifying these compounds, the development of an off-target cell detection system based on extended culture (Fig. 4) is worth highlighting. This detection system, in which putative PMSCs were successfully exposed in vitro by adding growth factors that were effective for the population while promoting the maturation of the PMSC origins via extended culture, was the core of the present study (Fig. 4 *A*–*C*). Furthermore, comprehensive gene expression data verified that the putative PMSCs were almost equivalent to in vivo PMSCs (Fig. 4 *D*–*G*), leading to the identification of effective compounds for in vivo PMSC removal. We expect that these methods will be effective not only for PMSC removal but also for the removal of off-target cells contaminating other types of PSC-derived products.

Docetaxel was not only the most potent agent for eliminating PMSCs but also reduced cystic structures composed of pancreatic duct-like cells to undetectable levels in vivo and in vitro (Figs. 5*E* and 6*K*). According to a previous report, nocodazole, which inhibits microtubule polymerization, the opposite mechanism of action to docetaxel, promotes the induction of pancreatic duct-like cells (32). Therefore, docetaxel may exhibit cyst removal effects by inhibiting differentiation into the pancreatic duct, independent of its PMSC removal activity. Although both in vivo PMSCs and cysts are presumed to be derived from nonendocrine progenitor cells (10) (Fig. 3*G*), the origin of PMSCs and cysts may not be completely identical based on the following observations: i) the ratio of putative PMSCs (PDX1^−^/CHGA^−^) and putative cysts (PDX1^+^/CHGA^−^) after extended culture differed between experiments (Figs. 4*B* and 5 *B* and *D*); and ii) the putative PMSCs and putative cysts showed different reduction ratios after cisplatin treatment (Fig. 5*E*). Previously, we reported that the nonendocrine progenitor population included multiple cell types (10). However, further studies are warranted to determine the differences between in vivo PMSC and cyst origins.

Identifying and resolving safety issues are important for the future expansion of cell therapies using PSCs. The findings of this study will significantly accelerate the realization of a cure for diabetes through cell therapy. Furthermore, the increase in the sensitivity and speed of detection of off-target cells via extended culture can be widely applied in the field of cell therapy.

## Materials and Methods

### Cell Culture and Differentiation.

Details for all cell culture and differentiation procedures can be found in *SI Appendix*. Ff-I14s04 and QHJI14s04 were provided by the CiRA Foundation of Kyoto University. Ff-I14s04 was derived from the same clone as QHJI14s04 but was cultured and stocked for nonclinical use. QHJI14s04 was created as the stock for clinical use. The use of human iPSCs was approved by the Ethical Review Committees of Kyoto University and Takeda Pharmaceutical Company Limited.

### Type 1 Diabetes Mouse Model.

Details for type 1 diabetes mouse model induction can be found in *SI Appendix*. All animal studies were conducted at Shonan iPark, an AAALAC international accreditation facility, and approved by the iPark Institutional Animal Care and Use Committee. All experiments were performed in accordance with the relevant guidelines and regulations, including the ARRIVE guidelines.

### Implantation and In Vivo Assessment.

Details for all implantation studies can be found in *SI Appendix*. Differentiated s7-iPIC or s6-iPIC aggregates were implanted in the subcutaneous space of anesthetized streptozotocin-injected NOD-scid mice or control NOD-scid mice (2.0 to 4.5 × 10^6^ cells/mouse).

### Tissue Processing and Immunostaining.

Details for all tissue processing and immunostaining can be found in *SI Appendix*. To determine the presence or absence of PMSCs and cysts, we prepared paraffin or frozen sections to ensure that the cross-sectional area of the graft was as large as possible and performed pathological and morphological determination by HE (Hematoxylin and eosin) staining. Considering that PMSCs and cysts have characteristic structures, they can be determined by HE staining. However, in cases such as docetaxel-treated s7-iPICs, where neither PMSCs nor cysts were present or could not be determined by HE staining alone, we performed a definitive diagnosis using immunostaining (insulin, glucagon, PDX1, HuN, Ki67, αSMA, and CK19). We determined that PMSCs and cysts were not present if the following three conditions were met: i) αSMA- and CK19-positive areas were absent, ii) areas with a high percentage of Ki67-positive cells were absent, and iii) most HuN-positive areas comprised PDX1^+^/insulin^+^/glucagon^+^ cells.

### scRNA-seq Library Preparation, Sequencing, and Data Processing.

Details for all scRNA-seq library preparation, sequencing, and data processing can be found in *SI Appendix*. A total of seven samples (one sample of Vitro s6-iPICs, two samples of Vivo s6-iPICs, one sample of reference human islets, one sample of Vitro s6-iPICs cultured without PD-166866, and two samples of s6-iPICs after 4 wk of extended culture, including EGF treatment) underwent scRNA-seq. We also obtained previously reported scRNA-seq data using in vivo grafts of ESC/iPSC-derived islet-like cells from publicly available database (GSE151117), including the UMI count matrices from three ESC (HUES8)-derived islet-like cell grafts (GSM4567001, GSM4567002, and GSM4567003) and two iPSC (WS4^corr^)-derived islet-like cell grafts (GSM4567004 and GSM4567005).

### Extended Culture.

Details for all extended cultures can be found in *SI Appendix*. The induced s6-iPICs were cultured for 4 wk in basic medium with or without EGF.

### Flow Cytometry.

Details for all flow cytometry analyses can be found in *SI Appendix*. The primary antibodies are listed in *SI Appendix*, Table S1.

### Statistical Analysis.

Data are expressed as the mean and SD values. Dunnett’s multiple-comparison test was performed based on the results of the homogeneity of variance test (Bartlett’s test) at a significance level of *P* < 0.05, as shown in Fig. 5 *C* and *E* and *SI Appendix*, Fig. S10*B*. Additionally, the Aspin–Welch test was performed at a significance level of *P* < 0.05 to determine the statistical significance between the two groups (Figs. 1*G* and 5*E*). As shown in Fig. 6*K*, Fisher’s exact test was performed for each of the two prioritized tiers (Tier 1: s7-iPICs vs. docetaxel-treated s7-iPICs; Tier 2: s7-iPICs vs. docetaxel-treated s6-iPICs), and adjustment for multiple testing was performed using the Bonferroni method. Since evaluation at single-cell resolution showed no prominent batch effect in our differentiation protocol (10), the in vivo implantation data from multiple induction batches were combined for statistical analysis (Figs. 1*G* and 6*K*). All statistical analyses were performed using the Statistical Analysis System version 9.3 (SAS Institute, NC).

## Supplementary Material

Appendix 01 (PDF)

## Data Availability

Single-cell RNA sequencing data were deposited in the Gene Expression Omnibus database (GSE213617) (33). The computer code will be deposited in the GitHub.
