# Supplementary material for: Identification and removal of unexpected proliferative off-target cells emerging after iPSC-derived pancreatic islet cell implantation
Source: Proc Natl Acad Sci U S A. 2024 Apr 10;121(16):e2320883121. doi: 10.1073/pnas.2320883121 (PMC11032438; doi:10.1073/pnas.2320883121)
Supplement: Supplementary file 1 — Appendix 01 (PDF) [file pnas.2320883121.sapp.pdf]

PNAS

Supporting Information for

Identification and removal of unexpected proliferative off-target cells emerging after iPSC-derived pancreatic islet cell implantation

Hideyuki Hiyoshi[1,2,*], Kensuke Sakuma[1,2], Shinya Asano[3], Stephanie C. Napier[1,2], Shuhei Konagaya[2,4], Taisuke Mochida[1,2], Hikaru Ueno[1,2], Takeshi Watanabe[5], Yoshiaki Kassai[1,2], Hirokazu Matsumoto[1,2], Ryo Ito[1,2] and Taro Toyoda[2,4,6,*]

*Co-corresponding authors

Hideyuki Hiyoshi, DVM

E-mail: hideyuki.hiyoshi@takeda.com

Taro Toyoda, PhD

E-mail: t.toyoda@cira.kyoto-u.ac.jp

This PDF file includes:

Supporting Text

SI References

Figures S1 to S12

Table S1

Supporting Information Text

Supplemental Materials and Methods

Cell culture and differentiation

Cells were maintained on iMatrix-511 (Nippi)-coated dishes in StemFit AK03N (Ajinomoto) at 37 °C in a humidified 5% CO_2 incubator. Cells were passaged every 3 or 4 days via non-enzymatic dissociation using 0.5 mM EDTA (Thermo Fisher Scientific) and subjected to differentiation experiments, usually after more than 2 weeks of running culture. For differentiation culture to generate s7-iPICs, we performed 3D stirred-floating aggregate culture based on our previous reports (1-3). The details of a typical differentiation culture are provided below.

Stage 1

Dissociated undifferentiated iPSCs were resuspended at a density of 2×10^5 cells/mL in AK03N medium containing 10 μM Y-27632 (FUJIFILM Wako). The cells were cultured in a spinner-type 30-mL bioreactor (ABLE Corporation & Biott) or a vertical mixing 0.25-L bioreactor (SATAKE MultiMix Corporation). The following day, the aggregated cells were cultured in DMEM (high glucose, GlutaMAX Supplement, pyruvate; Thermo Fisher Scientific) supplemented with 1% (v/v) penicillin/streptomycin (P/S, FUJIFILM Wako), 1× B-27 (#17504001 or A1895601; Thermo Fisher Scientific), 1% Pluronic® F-68 (Sigma-Aldrich) to reduce fluid-induced mechanical damage, 5–10 ng/mL activin A (PeproTech), 3 μM CHIR99021 (Axon Medchem), and 1% DMSO (FUJIFILM Wako). The following day, CHIR99021 was removed from the medium and the culture was continued for another 2 days.

Stage 2

The cells were cultured in MCDB 131 medium (Thermo Fisher Scientific) supplemented with 1% P/S, 0.5× B27, 1% Pluronic® F-68, 50 ng/mL keratinocyte growth factor (KGF, R&D Systems), 4.44 mM glucose (added to generate a final concentration of 10 mM, FUJIFILM Wako), 1.5 g/L $NaHCO_3$ (FUJIFILM Wako), and 1% GlutaMAX (Thermo Fisher Scientific) for 4 days.

Stage 3

Continuing culture was performed with improved MEM (iMEM, Thermo Fisher Scientific) containing 1% P/S, 0.5× B27, 1% Pluronic® F-68, 50 ng/mL KGF, 100 ng/mL Noggin (FUJIFILM Wako), 0.5 μM 3-keto-N-aminoethyl-N'-aminocaproyldihydrocinnamoyl cyclopamine (KAAD-cyclopamine, Toronto Research Chemicals), and 10 nM 4-[(E)-2-(5,6,7,8-tetrahydro- 5,5,8,8-tetramethyl-2-naphthalenyl)-1-propenyl] benzoic acid (TTNPB, Santa Cruz Biotechnology) for 3 days.

Stage 4

The cells were exposed to iMEM containing 1% P/S, 0.5× B27, 1% Pluronic® F-68, 100 ng/mL KGF, 50 ng/mL EGF (R&D Systems), 10 mM nicotinamide (STEMCELL Technologies), 0.1 μM TR05991851

(ROCK inhibitor, Takeda Pharmaceutical Company), 0.5 μM phorbol 12,13-dibutyrate (PdBU, Merck Millipore), and 5 ng/mL activin A for 4 days.

Stage 5

The cells were treated with iMEM with 1% P/S, 0.5× B27, 1% Pluronic® F-68, 0.25 μM SANT-1 (Merck Millipore), 50 nM retinoic acid (Merck Millipore), 10 μM ALK5 inhibitor II (Santa Cruz Biotechnology), 100 nM LDN-193189 (MedChemExpress), 1 μM L-3,3',5-triiodothyronine (T3, Merck Millipore), 50 ng/mL basic fibroblast growth factor (bFGF, PeproTech), 1 μM XAV939 (Merck Millipore), and 10 μM Y-27632 for 2 days.

Stage 6

The cells were cultured with iMEM containing 1% P/S, 0.5× B27, 1% Pluronic® F-68, 1 μM RO4929097 (γ-secretase inhibitor, "GSI" in Fig. 1A, Chem Scene), 10 μM ALK5 inhibitor II, 100 nM LDN-193189, and 1 μM T3 for 7 days. PD-166866 (1–10 μM, Merck Millipore) was added on day 4 of Stage 6. To generate s6-iPICs, cells were treated with the same medium for another 4 or 5 days.

Stage 7

Stage 7 medium was based on a previous report with our original modifications (2). The cells were exposed to MCDB 131 medium with 1% P/S, 2% fat-free BSA (FUJIFILM Wako), 14.44 mM glucose (added to generate a final concentration of 20 mM), 1.5 g/L $NaHCO_3$, 1% GlutaMAX, 0.5% ITS-X (Thermo Fisher Scientific), 10 μM ALK5 inhibitor II, 1 μM T3, 10 μM $ZnSO_4$ (Merck Millipore), 1.4 IU/mL heparin sodium salt (Nacalai Tesque), 1 mM N-acetyl cysteine (Merck Millipore), 10 μM Trolox (FUJIFILM Wako), 2 μM R428 (Selleck), 1–10 μM PD-166866, 3 μM TR06141363 (multi-kinase inhibitor, Takeda Pharmaceutical Company), and 10 μM Y-27632 for 4 or 5 days. To generate s7-iPICs for implantation, cells were dissociated and re-sized (1.0–1.4 × 10^3 cells/microwell) in an Elplasia microwell plate (Corning Incorporated) or a gas-permeable microwell culture bag (Toyo Seikan Group Holdings) at the start of Stage 7, and cells were then cultured statically until implantation. When inducing docetaxel-treated s7-iPICs, 1 μM docetaxel (FUJIFILM Wako) was added from day 4 of Stage 6.

Type 1 diabetes mouse model

NOD.CB17-Prkdc-scid/J (NOD-scid) mice were obtained from the Charles River. Male mice between the ages of 8 and 9 weeks were intraperitoneally administered multiple low doses of streptozotocin (50 mg/kg/day for 5 days; Sigma-Aldrich). Mice that became hyperglycemic within 2–3 weeks of streptozotocin injection were subjected to implantation experiments as a type 1 diabetes mouse model. Individual mice were implanted only once at one site over an extended period.

Implantation and *in vivo* assessment

Differentiated s7-iPIC or s6-iPIC aggregates were mixed with 100 μL of fibrinogen/50 μL of thrombin

solution, incubated at 37 °C for 5 min, and then implanted in the subcutaneous space of anesthetized streptozotocin-injected NOD-scid mice or control NOD-scid mice (2.0–4.5 × 10^6 cells/mouse). Fibrinogen from human plasma (Merck Millipore) and thrombin (Sigma) were reconstituted in iMEM and PBS to prepare 10 mg/mL and 50 IU/mL solutions, respectively, and stored at −80 °C until use. For implantation using alginate gel (Fig. 2A and B), cell aggregates were suspended in 3% alginate (NovaMatrix) in 25 mM HEPES buffer, cross-linked with 75 mM strontium chloride hexahydrate (FUJIFILM Wako) in 25 mM HEPES buffer, and formed into discs. We monitored the blood glucose levels of the implanted animals using the Accu-Chek Aviva system (Roche DC Japan) and collected plasma samples from the tail vein on the indicated days. For the oral glucose tolerance test, the mice were fasted overnight and orally injected with 2 g/kg glucose solution (Otsuka). Plasma samples were collected from the tail vein before and at 15, 30, 60, and 120 min after injection.

Tissue processing and immunostaining

Grafts were collected 23–30 weeks after implantation, fixed with 4% paraformaldehyde (FUJIFILM Wako) for over 24 h at 4 °C, and embedded in paraffin or frozen in OCT compound. Paraffin blocks were sectioned at 5 μm and used for hematoxylin and eosin (HE) staining and immunostaining. Frozen blocks were sectioned at 10 μm and used for immunofluorescence staining. The primary antibodies are listed in Table S1. Secondary antibodies were conjugated to Alexa Fluor 488, 546, or 568 (Thermo Fisher Scientific or Jackson ImmunoResearch). The sections were counterstained with Hoechst (Thermo Fisher Scientific) to label nuclei.

Single-cell RNA sequencing library preparation, sequencing, and data processing

A total of seven samples (one sample of Vitro s6-iPICs, two samples of Vivo s6-iPICs, one sample of reference human islets, one sample of Vitro s6-iPICs cultured without PD-166866, and two samples of s6-iPICs after 4 weeks of extended culture, including EGF treatment) underwent scRNA-seq. Vivo s6-iPIC samples were de-crosslinked using 100 mM sodium citrate solution. Human islets were purchased from PRODO (HP-18304-01, Donor age: 21 years, Donor sex: Male, Donor BMI: 27.3 kg/m^2, Donor HbA1c: 5.4%, Estimated purity: 85%, Estimated viability: 95%). Single-cell RNA-seq libraries were generated using the 10x Genomics Chromium™ controller and Chromium Single Cell 3 kit v2 (10x Genomics) according to the manufacturer's instructions. Successful cDNA amplification and library construction were ensured using high-sensitivity DNA kits on an Agilent 2100 Bioanalyzer (Agilent). The obtained libraries were sequenced using HiSeq (Illumina) with 150 bp paired-end reads at a depth of > 100,000 reads per cell. The sequencing reads of the *in vitro* s6-iPIC samples were aligned to the human GRCh38 genome reference, and gene counts were quantified as UMIs using Cell Ranger (10x Genomics). The sequencing reads of the *in vivo* s6-iPIC samples were first aligned to the human GRCh38 and mouse mm10 genome references. Cells with at least one UMI count of

the mouse genes were collected. Thereafter, the sequencing reads were aligned to the human GRCh38 genome reference and the collected cells containing mouse genes were removed for further analyses. We imported UMI count matrices into the R software Seurat package (4, 5), where normalization was performed according to the default settings. Cells with mitochondrial gene counts over 10% were regarded as dead or damaged and were removed before further analyses. The UMI count matrices were scaled by regressing the total number of UMI counts per cell and the percentage of mitochondrial gene counts. The genes for dimensional reduction were selected based on the average expression and dispersion of each gene, and a principal component analysis was performed. Principal components were used for Seurat's shared nearest neighbor graph clustering, and t-distributed stochastic neighbor embedding (*t*-SNE) or uniform manifold approximation and projection (UMAP) dimensional reduction were used to visualize the data. We examined the similarity between clusters using hierarchical clustering analysis of average expression. The cell cycle was evaluated and scored based on the expression of genes known as S-phase, G1, and G2M markers. To estimate cell types and similarities within s6-iPIC samples with reference to known tissues or cell lines, we performed RCA using the RCA package (6). Differential gene expression analysis of each cluster compared with the others was performed using the likelihood-ratio test for single-cell gene expression in the Seurat. For pseudotime analysis, the processed UMI count matrices were imported into a single-cell dataset for the monocle package (7-9). We selected the genes for ordering the cells using 'dpFeature' in monocle, to set Cluster 15 as the root (pseudotime was zero) and contracted the single-cell pseudotime via the 'DDRTree' algorithm. Differential gene expression analyses along with pseudotime were performed using the likelihood-ratio test in monocle, and genes with q-values less than 0.05 were identified as differentially expressed genes along with pseudotime. Differentially expressed genes were classified into gene clusters using pseudotime expression patterns, and functional enrichment analysis of each gene cluster was performed using the clusterProfiler package (10). We obtained previously reported scRNA-seq data using *in vivo* grafts of ESC/iPSC-derived islet-like cells from publicly available database (GSE151117). The UMI count matrices from three ESC (HUES8)-derived islet-like cell grafts (GSM4567001, GSM4567002, and GSM4567003) and two iPSC (WS4^{corr})-derived islet-like cell grafts (GSM4567004 and GSM4567005) were imported into Seurat package and low quality cells were removed. After normalization and scaling the data, the principal component analysis was performed and clustering and dimensional reduction were conducted.

Extended culture

The induced s6-iPICs were cultured for 4 weeks in MCDB 131 medium with 1% P/S, 2% fat-free BSA, 14.44 mM glucose (added to generate a final concentration of 20 mM), 1.5 g/L $NaHCO_3$, and 1% GlutaMAX, 0.5% ITS-X. We added 50 ng/mL EGF at the start of the extended culture as needed. The same extended culture was performed on cells treated with 2 μM R428, 1 mM N-acetylcysteine, 10

μM Trolox, 3 μM lenvatinib mesylate (Carbosynth Limited), 3 μM cisplatin (FUJIFILM Wako), 1 μM docetaxel, 10 μM cyclophosphamide monohydrate (FUJIFILM Wako), 1 μM tamoxifen citrate (Merck Millipore), 10 μM anastrozole (AstaTech), or 0.3 μM lapatinib ditosylate (LKT Laboratories, Inc.). These compounds were added from day 4 of Stage 6 until the end of the stage. The concentrations of each compound that did not affect s6-iPIC induction or cell number in prior experiments were selected.

Flow cytometry

Differentiation efficacy and quality were analyzed at the individual stages, based on developmental markers using immunostaining methods and LSRFortessa X20 flow cytometry equipment (BD), as described previously (2). The data were processed using FlowJo software. The primary antibodies are listed in Table S1. Secondary antibodies of the appropriate species were conjugated to Alexa Fluor 488, 546, 568, or 647 of the appropriate species (Thermo Fisher Scientific or Jackson).

Plasma glucose and hormone measurements

Plasma glucose, human C-peptide, and mouse C-peptide levels were measured using the Glucose Test C-II Wako (Fujifilm Wako), Mercodia Ultrasensitive C-peptide ELISA (Mercodia), and Mouse C-peptide Measurement Kit (Morinaga), respectively, according to the manufacturer's instructions.

SI References

1. Mochida T*, et al.* (2020) Insulin-Deficient Diabetic Condition Upregulates the Insulin-Secreting Capacity of Human Induced Pluripotent Stem Cell-Derived Pancreatic Endocrine Progenitor Cells After Implantation in Mice. *Diabetes* 69(4):634-646.
2. Hiyoshi H*, et al.* (2022) Characterization and reduction of non-endocrine cells accompanying islet-like endocrine cells differentiated from human iPSC. *Scientific reports* 12(1):4740.
3. Sakuma K*, et al.* (2023) CDK8/19 inhibition plays an important role in pancreatic β-cell induction from human iPSCs. *Stem cell research & therapy* 14(1):1.
4. Butler A, Hoffman P, Smibert P, Papalexi E, & Satija R (2018) Integrating single-cell transcriptomic data across different conditions, technologies, and species. *Nature biotechnology* 36(5):411-420.
5. Stuart T*, et al.* (2019) Comprehensive Integration of Single-Cell Data. *Cell* 177(7):1888-1902.e1821.
6. Li H*, et al.* (2017) Reference component analysis of single-cell transcriptomes elucidates cellular heterogeneity in human colorectal tumors. *Nature genetics* 49(5):708-718.
7. Trapnell C*, et al.* (2014) The dynamics and regulators of cell fate decisions are revealed by pseudotemporal ordering of single cells. *Nature biotechnology* 32(4):381-386.

8. Qiu X*, et al.* (2017) Single-cell mRNA quantification and differential analysis with Census. *Nature methods* 14(3):309-315.
9. Qiu X*, et al.* (2017) Reversed graph embedding resolves complex single-cell trajectories. *Nature methods* 14(10):979-982.
10. Yu G, Wang LG, Han Y, & He QY (2012) clusterProfiler: an R package for comparing biological themes among gene clusters. *Omics : a journal of integrative biology* 16(5):284-287.

Supplemental Figures

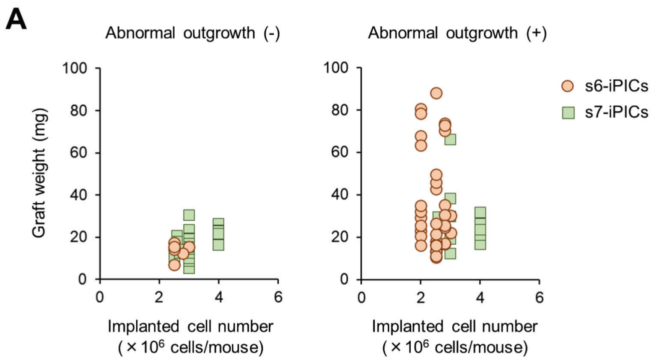

Fig. S1. Additional information for graft weight. Related to Fig. 1G.

(A) Individual graft weight distribution by type and number of implanted cells for grafts without abnormal outgrowth (s6-iPICs: n = 6, s7-iPICs: n = 76) and with abnormal outgrowth (s6-iPICs: n = 35, s7-iPICs: n = 12), respectively.

A

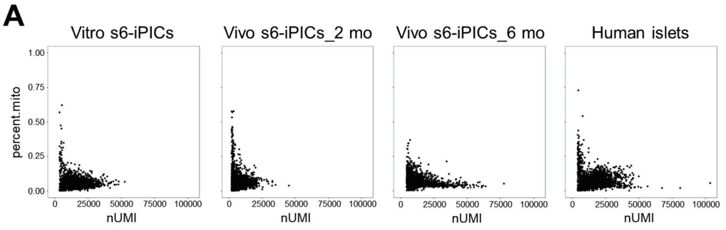

B

Sample ID	processing status	# of cells	median UMI counts per cell	median genes detected per cell
Vitro s6-iPICs	before QC	3125	13265	4014
	after QC	2936	13354.5	4036.5
Vivo s6-iPICs_2 months	before QC	2802	6130	2069
	exclude mouse cells	2755	6215	2084
	after QC	2422	6410	2122
Vivo s6-iPICs_6 months	before QC	1924	10455.5	2736.5
	exclude mouse cells	1912	10484	2741
	after QC	1584	11267.5	2832
Human islets	before QC	2959	14935	3400
	after QC	2419	14720	3410

C

Cluster No.	0	1	2	3	4	5	6	7	8	9	10	11	12	13	14	15	16	17	18	19	20
Vitro s6-iPICs	0	1015	813	721	0	0	1	0	0	0	202	172	0	0	0	0	1	0	11	0	0
Vivo s6-iPICs 2 months	1112	7	0	0	542	0	59	337	3	0	1	0	0	129	0	44	85	77	26	0	0
Vivo s6-iPICs 6 months	948	1	0	0	127	1	21	303	3	0	0	0	0	12	0	88	21	45	13	0	1
Human islets	0	0	0	0	0	645	564	0	505	239	0	0	161	0	135	0	18	0	33	69	50

D

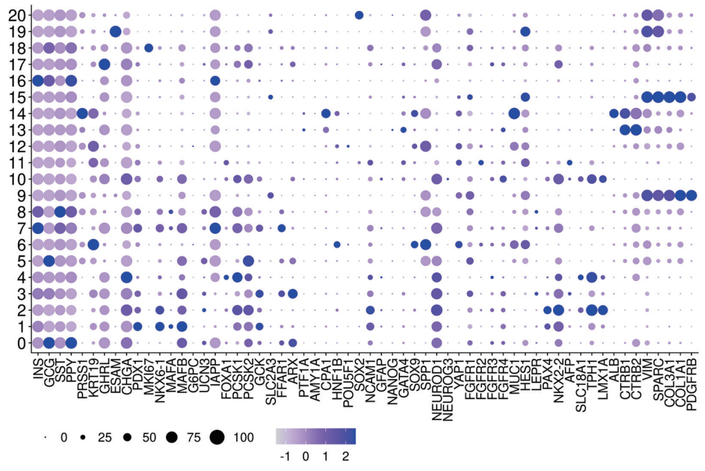

E

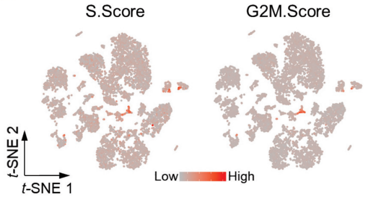

Fig. S2. Additional information for single-cell RNA sequencing analysis. Related to Fig. 2.

(A) Mitochondrial gene ratio and number of UMI counts for each individual cell in each sample.

(B) Number of cells, median UMI counts per cell, and median genes detected per cell before and after quality control (QC) by the mitochondrial gene ratio. For Vivo s6-iPIC samples, cells containing mouse genes were excluded prior to QC.

(C) Number of cells in the clusters classified in Fig. 2D. As Vivo s6-iPIC samples at 2 and 6 months post-implantation did not show any differences in cluster classification, we treated the two *in vivo* samples collectively.

(D) Bubble plot of endocrine and non-endocrine signature genes in the clusters classified in Fig. 2D. Color intensity indicates average relative expression levels. The bubble size indicates the percentage of expressing cells.

(E) Cell cycle phase assignments based on the S-, G2-, and M-phase gene signatures indicate a highly proliferative population (in red) within the *t*-SNE projection.

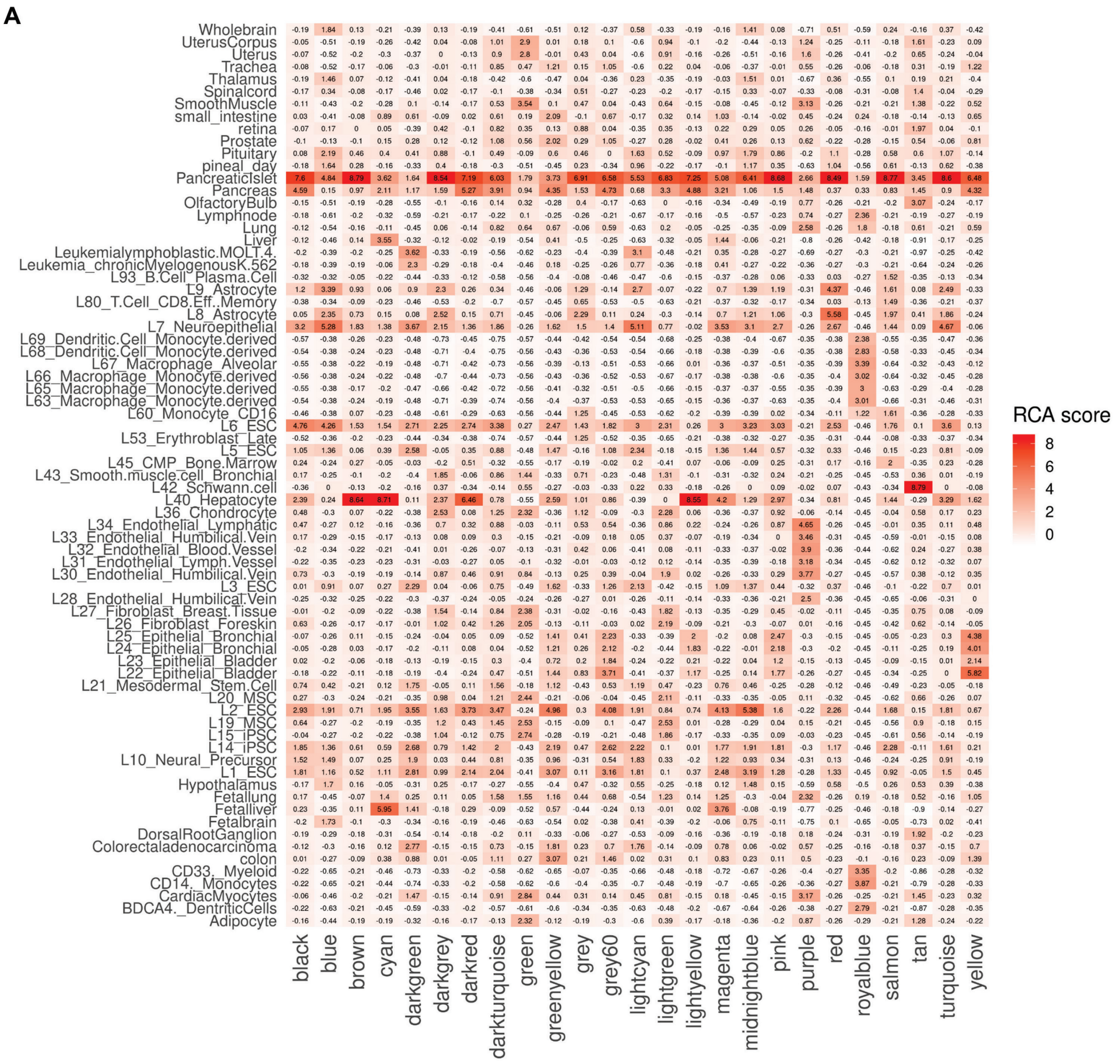

Fig. S3. Detailed information of RCA. Related to Fig. 3.

(A) Heatmap of similar tissues or cell lines for each color classified by the RCA in Fig. 3A.

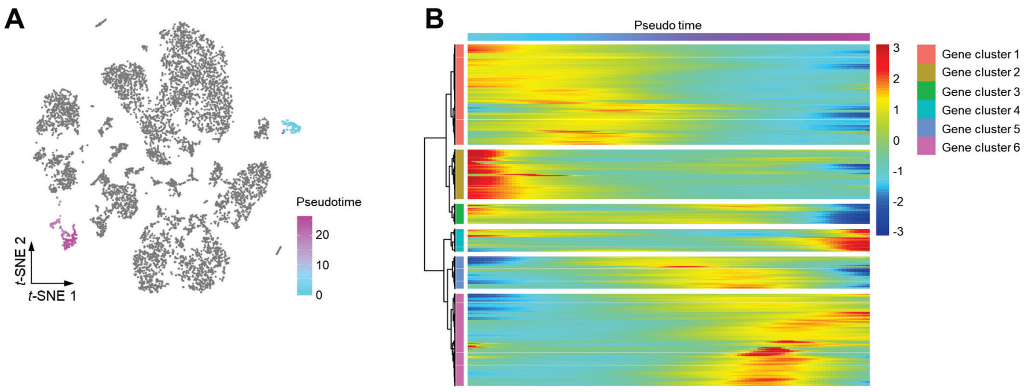

C

cluster	1	2	3	4	5	6
# of genes	1301	641	259	292	417	1198
top 10 significant genes (q < 0.05)	MGP, APOE, DLK1, IGF2, PPY, C7, SPARCL1, CHGA, FBLN1, MFAP4	ASPN, OGN, JUNB, IGF1, DPT, HOPX, NGFR, CAPN6, JUN, KLF2	RGS5, PLAT, APOC1, ACTB, CALM2, ANXA5, CYR61, ITM2B, UBC, BTG1	TIMP3, POSTN, CTHRC1, COL6A3, SCD, GREM1, COL1A2, FN1, CTSK, ARL4C	MMP1, IL24, TFPI2, GPX3, ADIRF, CHI3L1, CDH6, TPM1, MYL6, GDF15	MT2A, CXCL8, FABP4, IL11, CCL26, SPP1, SFRP2, CTSD, STC1, SOD2
# of GO terms	709	121	422	169	761	1276
top 10 significant GO terms (q < 0.05)	developmental process	regulation of transcription from RNA polymerase II promoter	positive regulation of ubiquitin-protein ligase activity involved in regulation of mitotic cell cycle transition	SRP-dependent cotranslational protein targeting to membrane	antigen processing and presentation of exogenous peptide antigen via MHC class I, TAP-dependent	immune system process
	anatomical structure development	transcription from RNA polymerase II promoter	positive regulation of proteolysis involved in cellular protein catabolic process	protein targeting to ER	antigen processing and presentation of exogenous peptide antigen via MHC class I	cellular response to chemical stimulus
	single-multicellular organism process	smoothened signaling pathway	positive regulation of cellular catabolic process	cotranslational protein targeting to membrane	antigen processing and presentation of peptide antigen via MHC class I	defense response
	single-organism developmental process	positive regulation of RNA metabolic process	positive regulation of ubiquitin protein ligase activity	establishment of protein localization to endoplasmic reticulum	response to organic substance	immune response
	multicellular organism development	positive regulation of gene expression	positive regulation of catabolic process	nuclear-transcribed mRNA catabolic process, nonsense-mediated decay	antigen processing and presentation	regulation of immune system process
	negative regulation of cellular process	positive regulation of RNA biosynthetic process	regulation of ubiquitin protein ligase activity	protein localization to endoplasmic reticulum	antigen processing and presentation of peptide antigen	response to stress
	system development	positive regulation of developmental process	positive regulation of cellular protein catabolic process	translational initiation	cellular response to organic substance	response to organic substance
	negative regulation of biological process	positive regulation of macromolecule biosynthetic process	negative regulation of ubiquitin-protein ligase activity involved in mitotic cell cycle	viral gene expression	cellular response to chemical stimulus	localization
	anatomical structure morphogenesis	positive regulation of transcription, DNA-templated	stimulatory C-type lectin receptor signaling pathway	viral transcription	antigen processing and presentation of exogenous peptide antigen	regulation of response to stimulus
	regulation of developmental process	positive regulation of nucleic acid-templated transcription	regulation of ubiquitin-protein ligase activity involved in mitotic cell cycle	nuclear-transcribed mRNA catabolic process	regulation of cellular amino acid metabolic process	cell migration

Fig. S4. Comparison of Clusters 15 and 9 using pseudotime analysis.

(A) Estimated pseudotime from Cluster 15 to Cluster 9 on the *t*-SNE projection.

(B) Expression heatmap of differentially expressed genes ordered by their common kinetics through pseudotime.

(C) Top 10 significant genes (*q*-value < 0.05) and enriched gene ontology (GO) terms (*q*-value < 0.05) of each gene cluster.

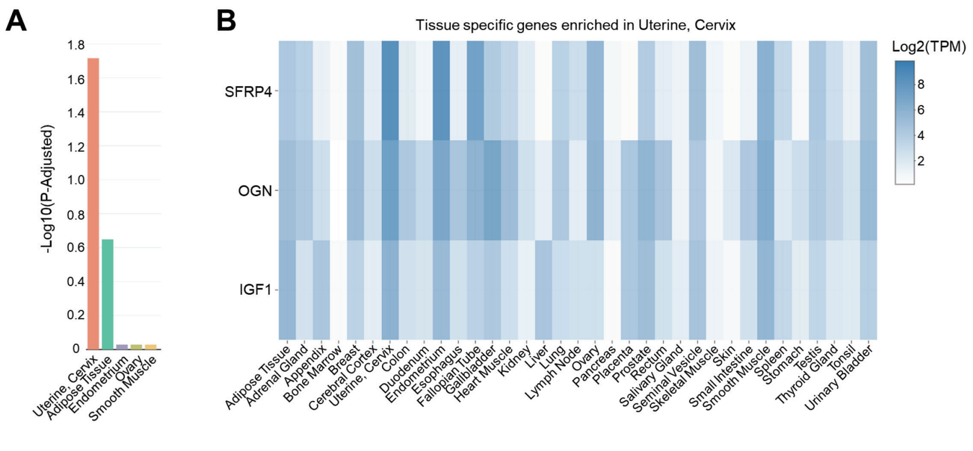

Fig. S5. Tissue-specific gene enrichment analysis of the Cluster 15 specific genes.

(A and B) Annotation of the top 20 differentially expressed genes in Cluster 15 (Fig. 3C) using TissueEnrich (https://tissueenrich.gdcb.iastate.edu/).

(A) Human tissues extracted as related tissues from the Human Protein Atlas dataset.

(B) Expression intensities of *SFRP4*, *OGN*, and *IGF1* (causative genes of high relevance to the uterus in Fig. S5A) in each human tissue.

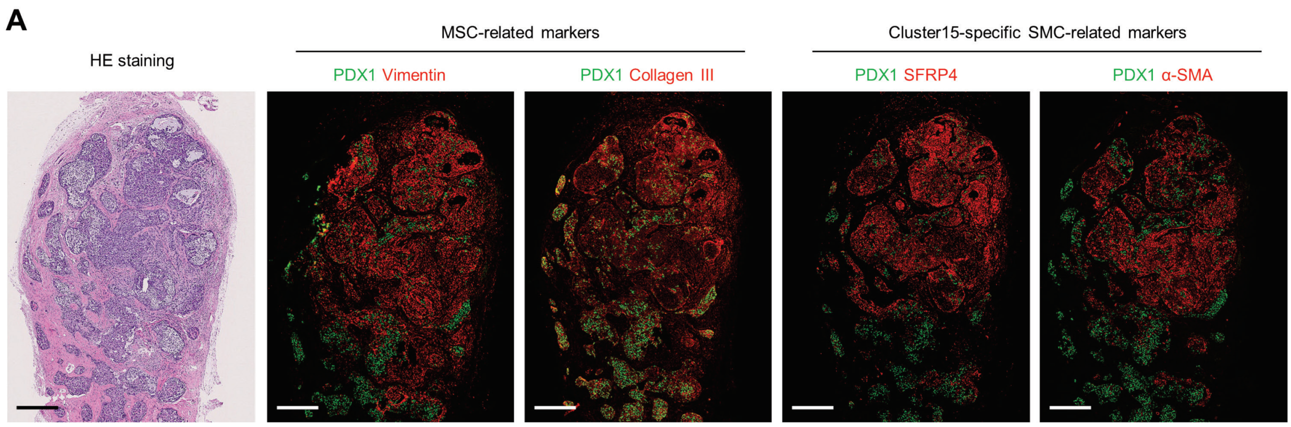

Fig. S6. Additional information for tissue immunostaining. Related to Fig. 3E and F.

(A) HE and immunohistochemical images of an s7-iPIC graft with abnormal outgrowth at 24 weeks post-implantation. Black and white scale bars indicate 500 μm. Images were taken from serial sections of the same sample as in Fig. 3E and F and are representative of dozens of samples showing similar results.

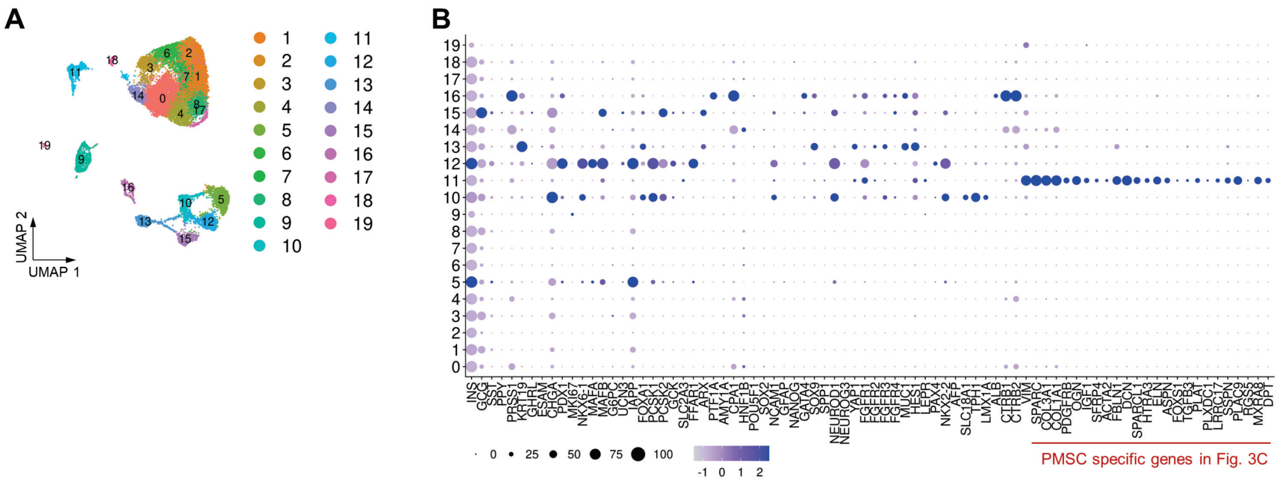

Fig. S7. Detection of PMSCs equivalent cells using previously reported scRNA-seq data of ESC/PSC-derived islet-like cell grafts.

(A) Shared nearest neighbors clustering on the UMAP projection using scRNA-seq data from *in vivo* samples included in GSE151117. We reanalyzed data from three ESC (HUES8)-derived islet-like cell grafts (GSM4567001, GSM4567002, and GSM4567003) and two iPSC (WS4^{corr})-derived islet-like cell grafts (GSM4567004 and GSM4567005).

(B) Bubble plot of endocrine and non-endocrine signature genes in the clusters classified in Fig. S7A. Cluster 11 expressed PMSC-specific markers such as *VIM*, *COL3A1*, *OGN*, *IGF1*, *SFRP4*, and *ACTA2*.

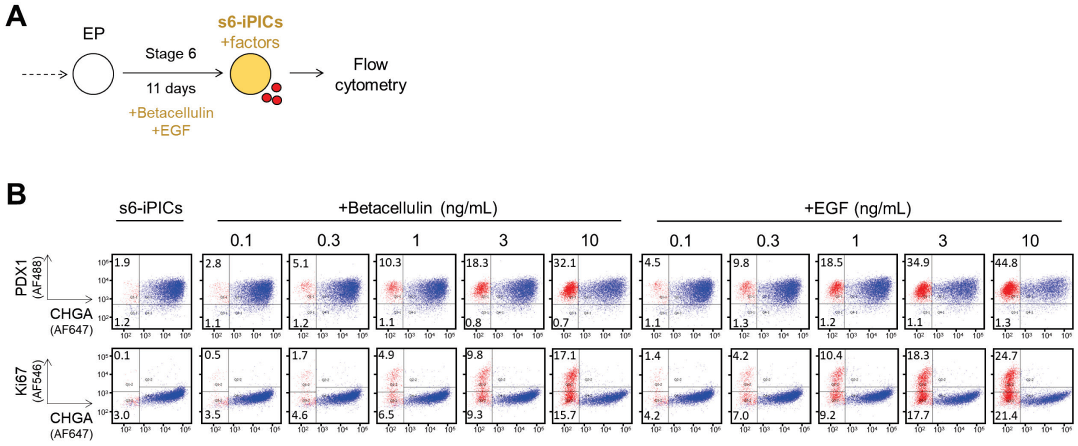

Fig. S8. Proliferation-promoting activity of betacellulin and EGF on the non-endocrine population in s6-iPICs.

(A) Schematic representation of s6-iPIC induction including betacellulin or EGF treatment, and subsequent flow cytometry analysis.

(B) Flow cytometry plots illustrating the protein expression of s6-iPICs differentiated with betacellulin or EGF treatment. The numbers in each plot diagram show the percentage of each population. The CHGA-negative non-endocrine population, shown in red, increased in a dose-dependent manner by betacellulin or EGF treatment.

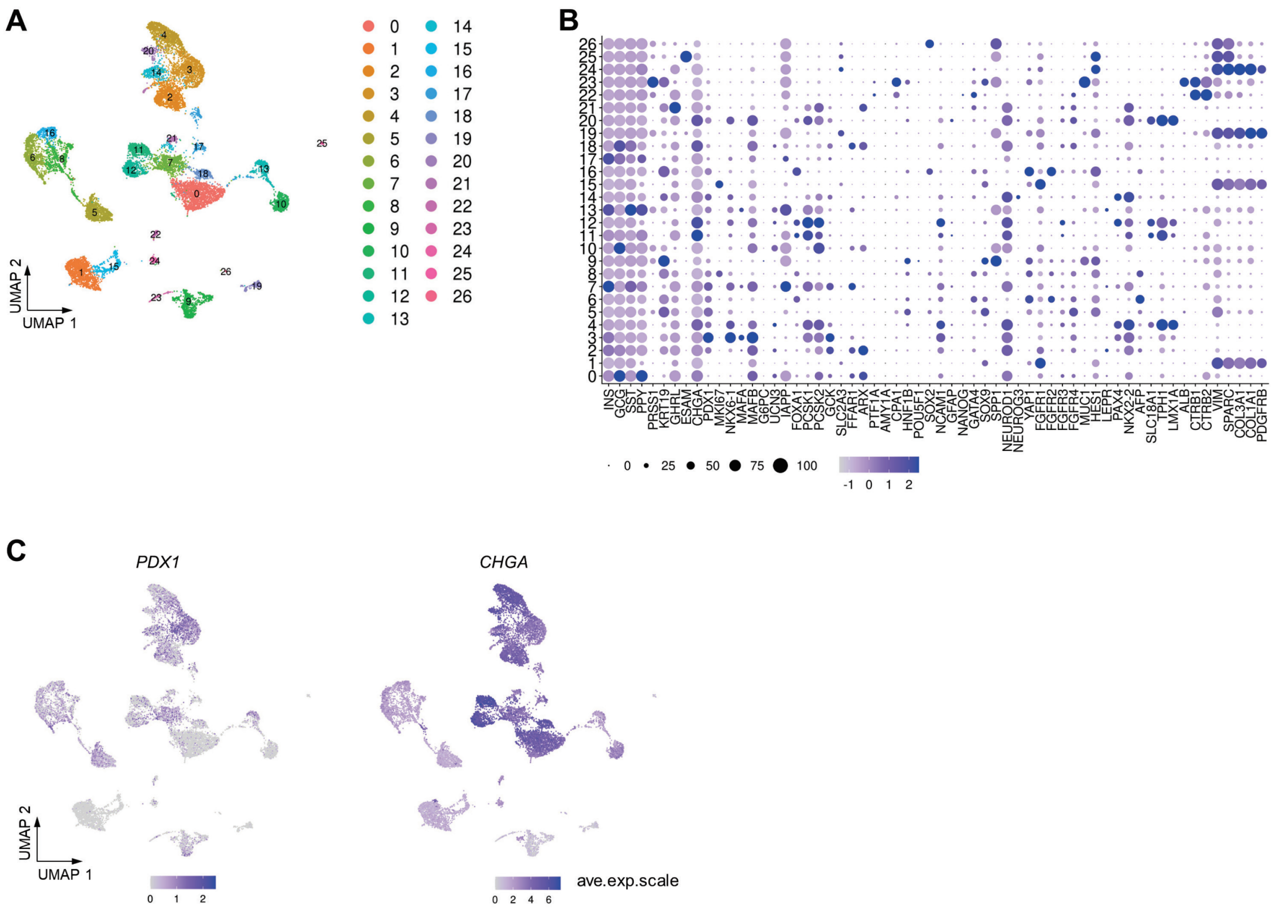
A
UMAP 2
UMAP 1
0 1 2 3 4 5 6 7 8 9 10 11 12 13 14 15 16 17 18 19 20 21 22 23 24 25 26
B
INS GCG SST PPY PRSS1 KRT19 GHRL ESAM CHGA PDX1 MKI67 NKX6-1 MAFA MAFB G6PC UCN3 IAPP FOXA1 PCSK1 PCSK2 GCK SLC2A3 FFAR1 ARX PTF1A AMY1A CPA1 HNF1B POU5F1 SOX2 NCAM1 GFAP NANOG GATA4 SOX9 SPP1 NEUROD1 NEUROG3 YAP1 FGFR1 FGFR2 FGFR3 FGFR4 MUC1 HES1 LEPR PAX4 NKX2-2 AFP SLC18A1 TPH1 LMX1A ALB CTRB1 CTRB2 VIM SPARC COL3A1 COL1A1 PDGFRB
0 25 50 75 100
-1 0 1 2
C
PDX1
CHGA
UMAP 2
UMAP 1
0 1 2
0 2 4 6
ave.exp.scale

D

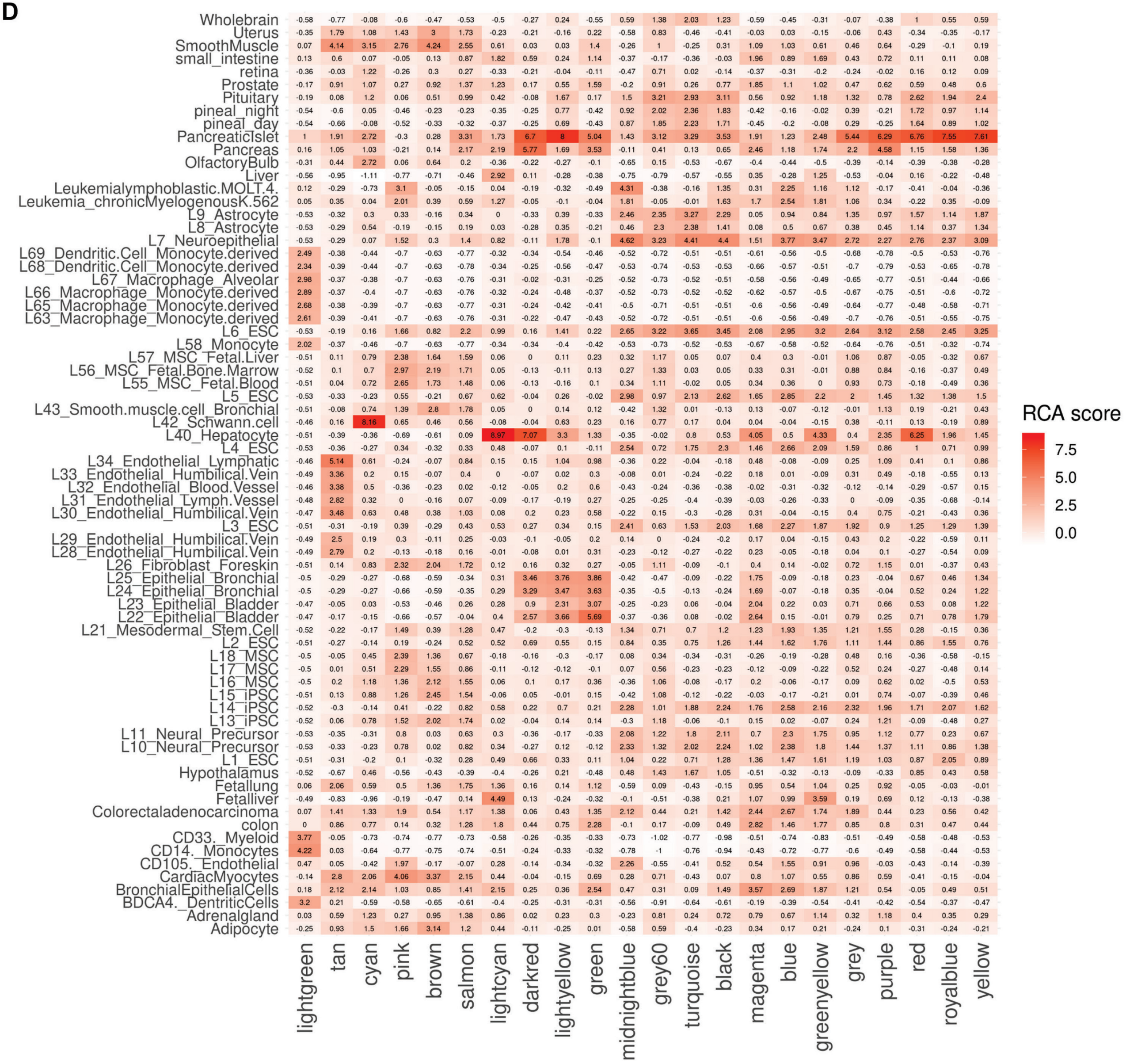

Fig. S9. Additional information for single-cell RNA sequencing analysis containing extended culture samples. Related to Fig. 4.

(A) Shared nearest neighbors clustering of the reanalyzed scRNA-seq data in Fig. 4D on the UMAP projection. Reanalysis with the additional samples re-assigned Clusters 15 and 9 in Fig. 2D to Clusters 24 and 19 in this figure, respectively.

(B) Bubble plot of endocrine and non-endocrine signature genes in the clusters classified in Fig. S9A. Color intensity indicates average relative expression levels. The bubble size indicates the percentage of expressing cells.

(C) Single-cell gene expression of *PDX1* and *CHGA* on the UMAP projection in Fig. 4D.

(D) Heatmap of similar tissues or cell lines for each color classified by the RCA in Fig. 4E.

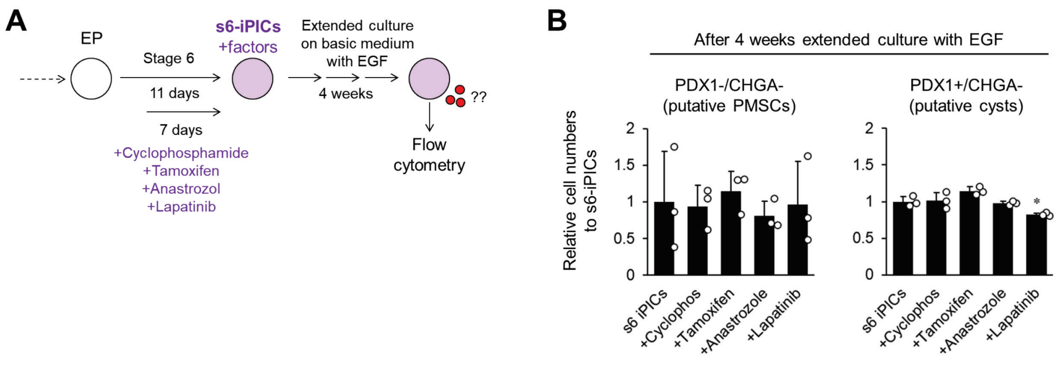

Fig. S10. Evaluation of the effects of cyclophosphamide, tamoxifen, anastrozole, and lapatinib treatment on putative PMSC and cyst populations.

(A) Schematic representation of s6-iPIC derivatives induced with or without additional compound treatment and subsequent extended culture and flow cytometry analysis.

(B) Relative cell numbers of PDX1$^-$/CHGA$^-$, PDX1$^+$/CHGA$^-$, and CHGA$^+$ populations in each s6-iPIC derivative post-extended culture. The number of cells in control s6-iPICs was set to "1". The number of cells in each population was calculated from flow cytometry results (percentage of each population) and live cell counts. Data are shown as the mean ± SD (n = 3, technical replicates). *$P < 0.05$, versus s6-iPICs, Dunnett's test.

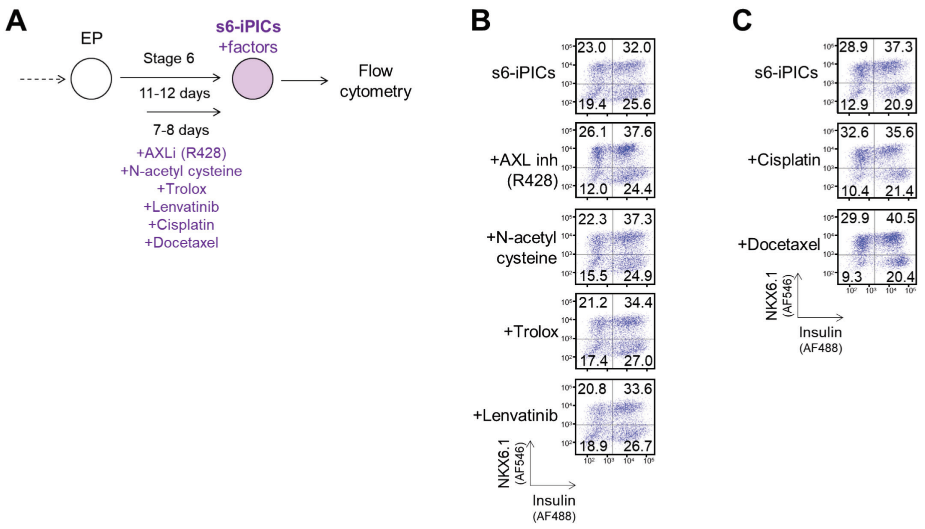

Fig. S11. Evaluation of the effect of kinase inhibitors, cisplatin, and docetaxel treatment on insulin-positive cell rates.

(A) Schematic representation of s6-iPIC derivatives induced with or without additional compound treatment and flow cytometry analysis.

(B and C) Representative flow cytometry plots illustrating insulin and NKX6.1 protein expression before an extended culture of s6-iPIC derivatives.

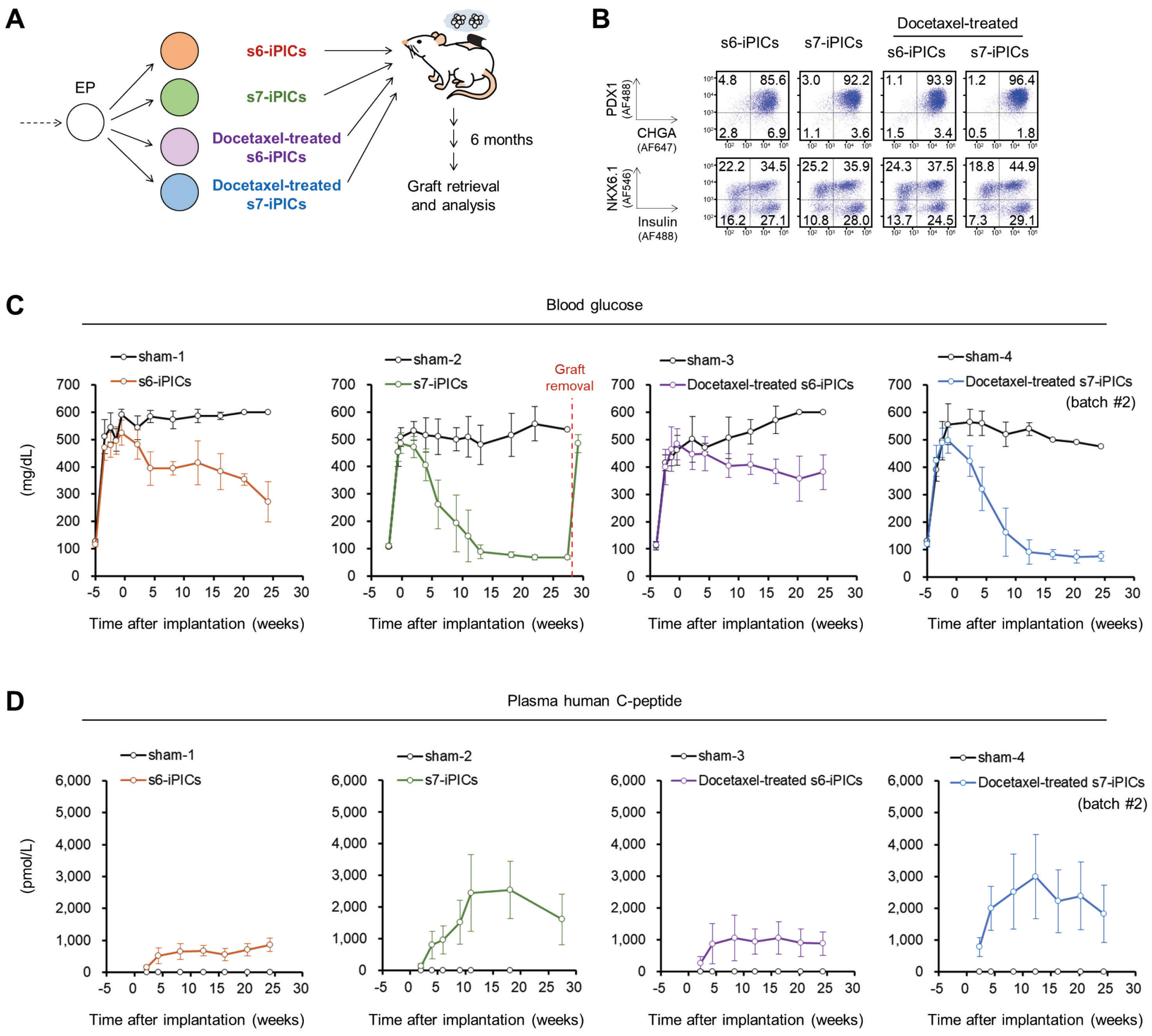
A
EP
s6-iPICs
s7-iPICs
Docetaxel-treated s6-iPICs
Docetaxel-treated s7-iPICs
6 months
Graft retrieval and analysis
B
Docetaxel-treated
s6-iPICs
s7-iPICs
s6-iPICs
s7-iPICs
PDX1 (AF488)
CHGA (AF647)
NKX6.1 (AF546)
Insulin (AF488)
4.8
85.6
2.8
6.9
3.0
92.2
1.1
3.6
1.1
93.9
1.5
3.4
1.2
96.4
0.5
1.8
22.2
34.5
16.2
27.1
25.2
35.9
10.8
28.0
24.3
37.5
13.7
24.5
18.8
44.9
7.3
29.1
C
Blood glucose
(mg/dL)
sham-1
s6-iPICs
sham-2
s7-iPICs
Graft removal
sham-3
Docetaxel-treated s6-iPICs
sham-4
Docetaxel-treated s7-iPICs
(batch #2)
Time after implantation (weeks)
D
Plasma human C-peptide
(pmol/L)
sham-1
s6-iPICs
sham-2
s7-iPICs
sham-3
Docetaxel-treated s6-iPICs
sham-4
Docetaxel-treated s7-iPICs
(batch #2)
Time after implantation (weeks)

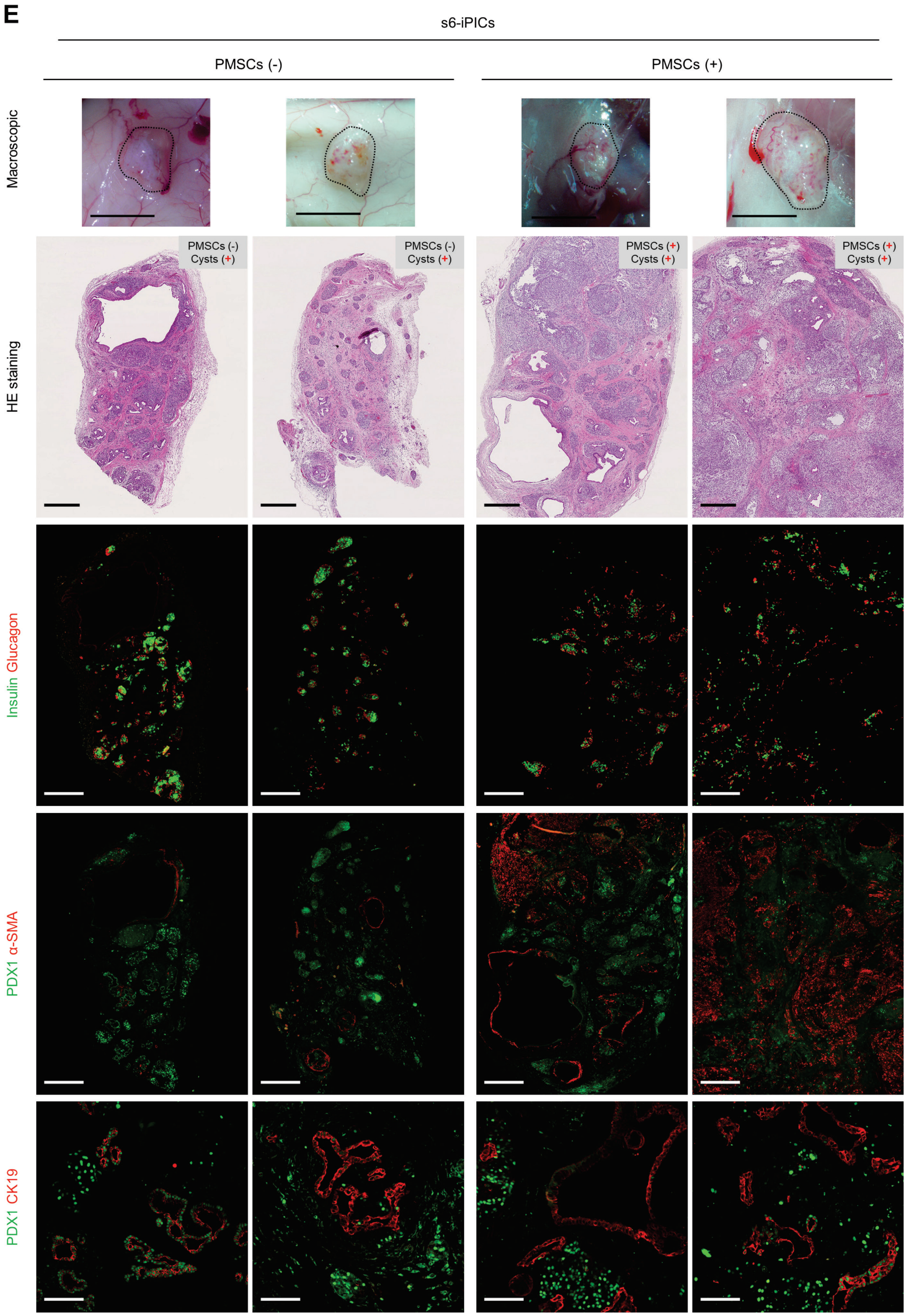
E
s6-iPICs
PMSCs (-)
PMSCs (+)
Macroscopic
HE staining
PMSCs (-)
Cysts (+)
PMSCs (+)
Cysts (+)
Insulin Glucagon
PDX1 α-SMA
PDX1 CK19

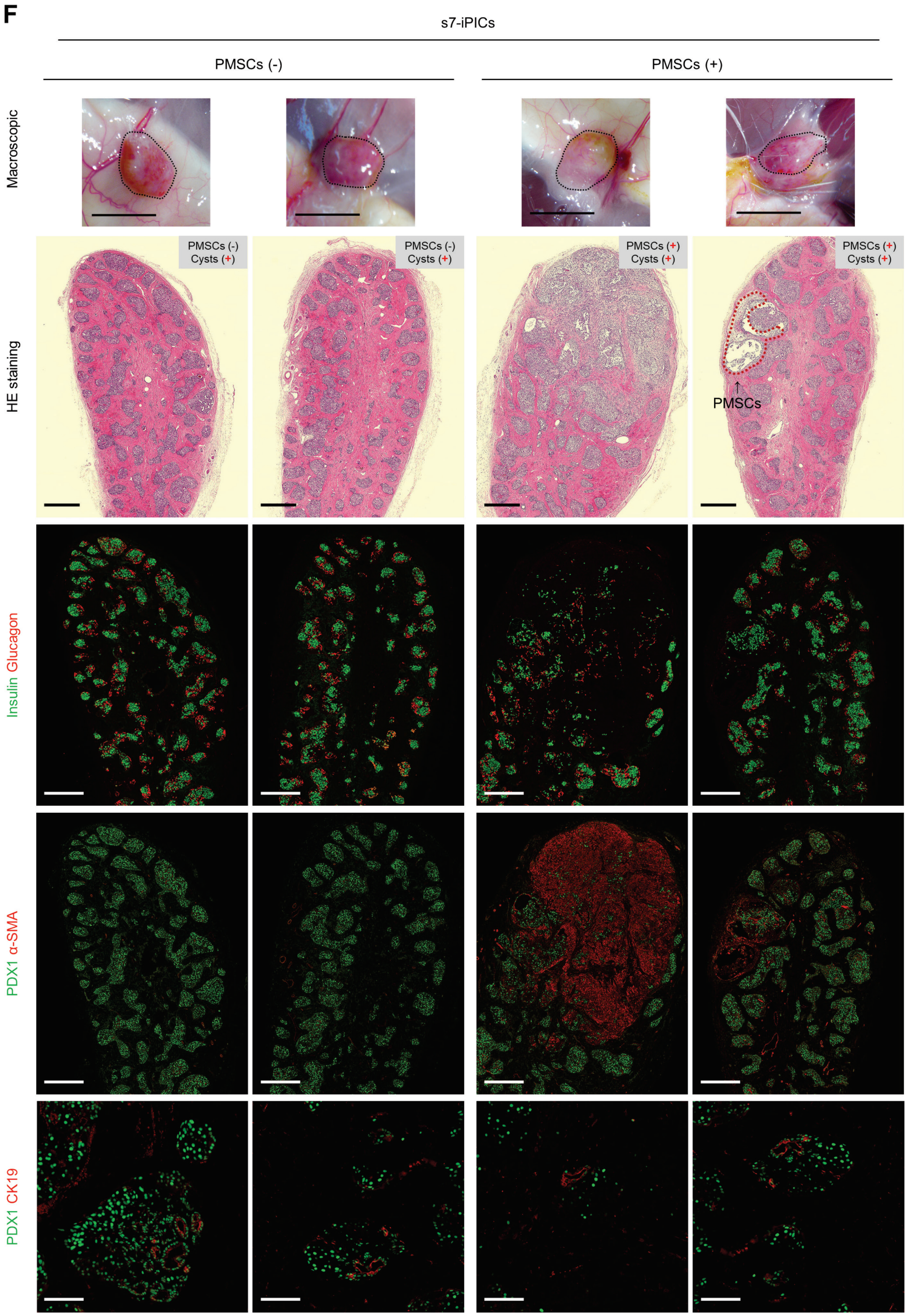
F
s7-iPICs
PMSCs (-)
PMSCs (+)
Macroscopic
HE staining
PMSCs (-)
Cysts (+)
PMSCs (-)
Cysts (+)
PMSCs (+)
Cysts (+)
PMSCs (+)
Cysts (+)
PMSCs
Insulin Glucagon
PDX1 α-SMA
PDX1 CK19

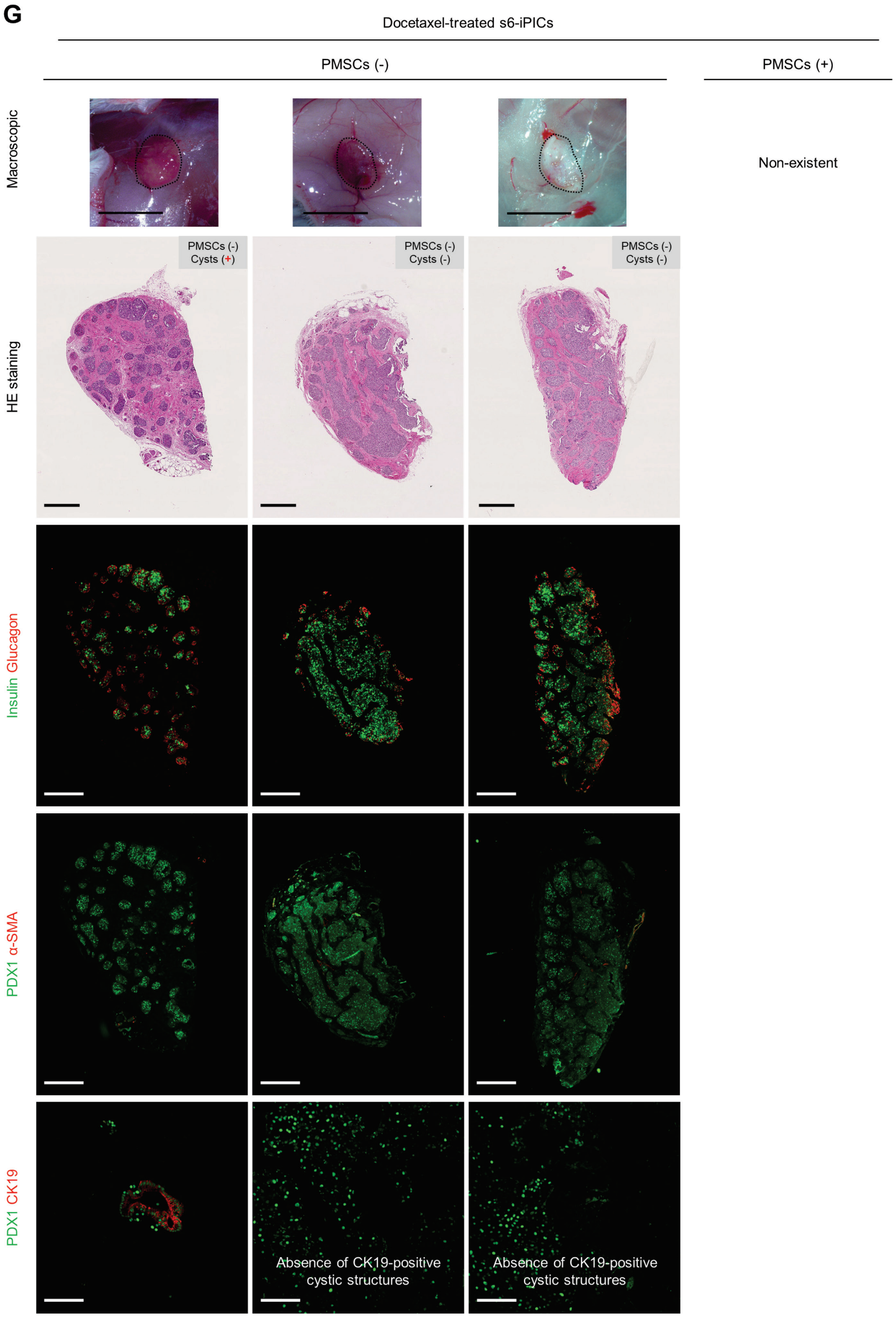
G
Docetaxel-treated s6-iPICs
PMSCs (-)
PMSCs (+)
Macroscopic
Non-existent
HE staining
PMSCs (-)
Cysts (+)
PMSCs (-)
Cysts (-)
PMSCs (-)
Cysts (-)
Insulin Glucagon
PDX1 α-SMA
PDX1 CK19
Absence of CK19-positive cystic structures
Absence of CK19-positive cystic structures

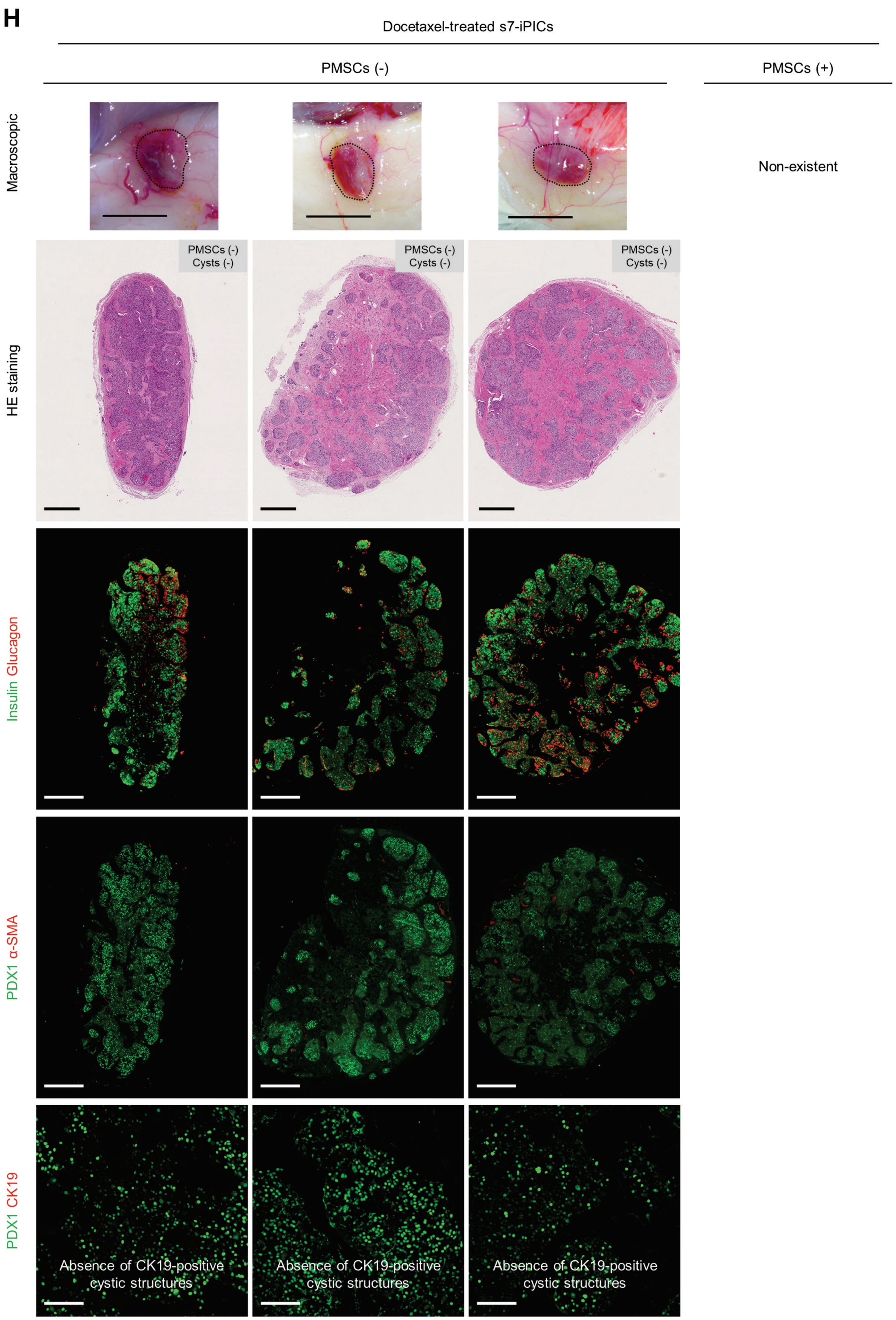
H
Docetaxel-treated s7-iPICs
PMSCs (-)
PMSCs (+)
Macroscopic
Non-existent
HE staining
PMSCs (-)
Cysts (-)
PMSCs (-)
Cysts (-)
PMSCs (-)
Cysts (-)
Insulin Glucagon
PDX1 α-SMA
PDX1 CK19
Absence of CK19-positive cystic structures
Absence of CK19-positive cystic structures
Absence of CK19-positive cystic structures

Fig. S12. Additional information for implantation studies. Related to Fig. 6.

(A) Schematic representation of four types of iPICs differentiation and subcutaneous implantation using fibrin gel.

(B) Representative flow cytometry plots illustrating protein expression before implantation of four types of iPICs. The numbers in each plot diagram show the percentage of each population.

(C and D) Blood glucose and plasma human C-peptide levels after implantation of s6-iPICs (3.0 × 10^6 cells/mouse), s7-iPICs (3.0 × 10^6 cells/mouse), docetaxel-treated s6-iPICs (4.0 × 10^6 cells/mouse), and docetaxel-treated s7-iPICs (batch #2) (4.0 × 10^6 cells/mouse). Data are shown as mean ± SD (sham-1: n = 5→1, s6-iPICs: n = 5→3, sham-2: n = 5→2, s7-iPICs: n = 16→6, sham-3: n = 5→1, docetaxel-treated s6-iPICs: n = 11→8, sham-4: n = 5→1, docetaxel-treated s7-iPICs (batch #2): n = 22→17). The decrease in the n number was caused by unexpected death.

(E–H) Macroscopic, HE, and immunohistochemical images of s6-iPIC grafts **(E)**, s7-iPIC grafts **(F)**, docetaxel-treated s6-iPIC grafts **(G)**, and docetaxel-treated s7-iPICs grafts **(H)**. In the macroscopic images, the areas surrounded by dotted lines indicate areas where the implanted cells engrafted subcutaneously. Black and white scale bars indicate 5 mm in macroscopic images, 500 μm in HE, insulin/glucagon, PDX1/α-SMA images, and 100 μm in PDX1/CK19 images.

Supplemental Table

Table S1. List of primary antibodies used for immunofluorescence staining.

Antigen	Species	Manufacturer	Catalog #	Clonality	Dilution for immunohistochemistry	Dilution for flow cytometry
INS	Rabbit	CST	3014	Monoclonal (C27C9)	1 : 200	-
INS	Rat	DSHB	GN-ID4	Monoclonal (GN-ID4)	-	1 : 600
GCG	Mouse	Sigma	G2654	Monoclonal (K79bB10)	1 : 200	-
PDX1	Goat	R&D systems	AF2419	Polyclonal	1 : 200	1 : 200
NKX6.1	Rabbit	CST	54551S	Monoclonal (D8O4R)	-	1 : 600
CK19	Mouse	Dako	M0888	Monoclonal (RCK108)	1 : 200	
Ki67	Rabbit	CST	9129	Monoclonal (D3B5)	1 : 100	-
Ki67	Mouse	BD Biosciences	556003	Monoclonal (B56)	-	1 : 100
HuN	Mouse	Takara Bio	Y40400	Monoclonal	1 : 100	-
IGF1	Mouse	Merck Millipore	05-172	Monoclonal (Sm1.2)	1 : 100	-
α-SMA	Mouse	Abcam	ab7817	Monoclonal (1A4)	1 : 500	1 : 6000
Vimentin	Rabbit	CST	5741S	Monoclonal (D21H3)	1 : 100	-
Collagen Ⅲ	Rabbit	Abcam	ab7778	Polyclonal	1 : 50	-
SFRP4	Rabbit	Abcam	ab154167	Monoclonal (EPR9389)	1 : 50	-
CHGA	Rabbit	Abcam	ab68271	Monoclonal (EP1030Y)	-	1 : 500
